# Leptin Aggravates Thoracic Aortic Dissection Through Impairment of Energy Metabolism in Nrip2^+^ Smooth Muscle Cells

**DOI:** 10.1002/advs.202502027

**Published:** 2025-07-16

**Authors:** Ling Chen, Yujie Zhu, Xi Yang, JiangBin Wu, Keyuan Chen, Wuqing Huang, Lu Fang, Qi Zhang, Jie Chen, Jianing Gao, Huanhuan Cao, Meifang Wu, Zhihuang Qiu, Yuling Zhang, Yue Shen, Qiuyu Huang, Zhiyong Lin, Lemin Zheng, Liangwan Chen

**Affiliations:** ^1^ Department of Cardiovascular Surgery Fujian Medical University Union Hospital Fuzhou 350100 China; ^2^ Key Laboratory of Cardio‐Thoracic Surgery (Fujian Medical University) Fujian Province University Fuzhou 350001 China; ^3^ The Institute of Cardiovascular Sciences and Institute of Systems Biomedicine School of Basic Medical Sciences State Key Laboratory of Vascular Homeostasis and Remodeling NHC Key Laboratory of Cardiovascular Molecular Biology and Regulatory Peptides Beijing Key Laboratory of Cardiovascular Receptors Research Health Science Center Peking University Beijing 100191 China; ^4^ Department of Epidemiology and Health Statistics School of Public Health Fujian Medical University Fuzhou Fujian 350004 China; ^5^ Fujian Provincial Center for Cardiovascular Medicine Fuzhou 350100 China; ^6^ Beijing Tiantan Hospital China National Clinical Research Center for Neurological Diseases Advanced Innovation Center for Human Brain Protection Beijing Institute of Brain Disorders The Capital Medical University Beijing 100050 China; ^7^ Cardiology Division Emory University School of Medicine 100 Woodruff Circle Atlanta GA 30322 USA; ^8^ Department of Cardiovascular Affiliated Hospital of Putian University Putian Fujian 351100 China

**Keywords:** energy metabolism, leptin, nuclear receptor interacting protein 2 (Nrip2), phenotypic transitions, thoracic aortic dissection (TAD)

## Abstract

Obesity is a significant risk factor for thoracic aortic dissection (TAD), as supported by UK Biobank data showing obese individuals have a higher risk of TAD. The study investigates leptin, a hormone elevated in obesity, and finds that hyperleptinemia is common in TAD patients, suggesting its role in disease pathogenesis. Using leptin‐knockout mice, it is demonstrated that exogenous leptin exacerbates TAD by promoting phenotypic transitions in vascular smooth muscle cells (VSMCs). These adverse effects are reversible with pharmacologic leptin blockade, indicating potential therapeutic benefits. Single‐cell RNA sequencing reveals a novel smooth muscle cells (SMC) cluster, Nrip2^+^, in the aorta, with a distinct contractile gene profile. Increased Nrip2^+^ VSMCs are linked to enhanced mitochondrial energy metabolism. Elevated Nrip2^+^ VSMCs inhibit the leptin‐induced transition from a contractile to a synthetic phenotype, reducing TAD incidence. The findings suggest that high blood leptin levels contribute to the increased TAD risk in obese individuals by suppressing Nrip2^+^ SMCs, leading to abnormal mitochondrial metabolism and VSMC phenotypic transitions. Thus, targeting leptin to boost Nrip2^+^ VSMC metabolic activity is a promising strategy for TAD prevention and treatment.

## Graphical abstract

Epidemiological data confirm obesity elevates thoracic aortic dissection (TAD) risk. Patient analyses implicate hyperleptinemia, where leptin binding to vascular smooth muscle cell (VSMC) receptors triggers Socs3‐mediated suppression of Stat5a transcriptional activity. This attenuated Stat5a binding weakens Nrip2 promoter activation, impairing mitochondrial metabolism and inducing mitochondrial dysfunction. Consequently, VSMCs shift from a contractile to a synthetic phenotype, propelling TAD development and progression.

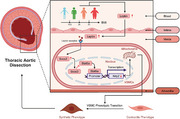

## 1 Introduction

Thoracic aortic dissection (TAD), a life‐threatening condition involving rupture of the aortic intima, carries significant mortality without intervention.^[^ ^1^, ^2^, ^3^ ^]^ Its pathogenesis centers on medial degeneration within the aortic wall, driven by chronic risk factors like hypertension and smoking.^[^ ^4^ ^]^ This degeneration, characterized by structural abnormalities and inflammatory lesions, initiates a cascade: intravascular pressure surges promote aortic dilation and smooth muscle cell (SMC) apoptosis.^[^ ^5^ ^]^ Apoptotic cells activate inflammasomes, releasing pro‐inflammatory factors that recruit inflammatory cells.^[^ ^6^ ^]^ Infiltrating neutrophils and macrophages further secrete cytokines and matrix metalloproteinases (MMPs), accelerating elastic fiber degradation and, crucially driving the phenotypic transitions of vascular SMCs (VSMCs) from a contractile to a synthetic state.^[^ ^7^, ^8^ ^]^ This switch compromises aortic wall contractility, enabling progressive dilation and ultimately, intimal rupture.

Obesity is a major cardiovascular risk factor strongly linked to hypertension and diabetes, conditions prevalent in TAD patients.^[^ ^9^ ^]^ Epidemiological data robustly associate elevated Body Mass Index (BMI) with increased all‐cause mortality and, as our UK Biobank (UKB) analysis confirms, specifically with heightened TAD risk.^[^ ^10^, ^11^, ^12^, ^13^, ^14^ ^]^ While the obesity‐TAD link is established, the underlying molecular mechanisms remain incompletely defined. Serum analysis of TAD patients revealed significant hyperleptinemia, particularly in obese individuals. Leptin, an adipokine regulating energy homeostasis, is also implicated in vascular pathologies, including injury, oxidative stress, and thrombosis.^[^ ^15^, ^16^ ^]^ Critically, although hyperleptinemia is recognized in obesity, its specific role in mediating obesity‐associated TAD risk—particularly concerning VSMC phenotypic transitions and energy metabolism dysregulation—represents a significant gap in understanding. We therefore hypothesize that hyperleptinemia acts as a key mechanistic link between obesity and TAD pathogenesis, primarily by impairing VSMC energy metabolism and driving the detrimental contractile‐to‐synthetic phenotypic transition. Consequently, targeting leptin signaling emerges as a promising therapeutic strategy to mitigate TAD progression. This study specifically investigates the leptin axis in VSMC phenotypic regulation and its contribution to TAD development.

## 2 Results

### 2.1 Hyperleptinemia: A Putative Association Between Obesity and Elevated Thoracic Aortic Dissection Risk

**Figure 1:**
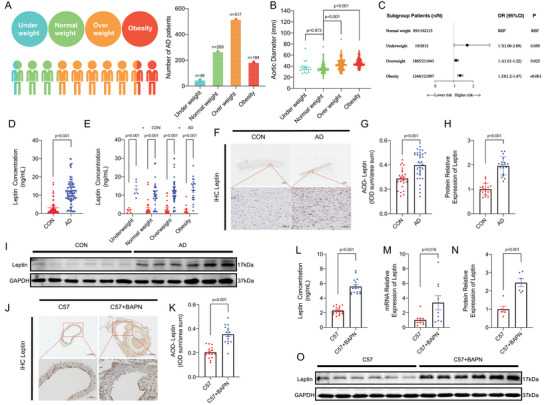
Overweight and Obese Populations are More Likely to Develop Thoracic Aortic Dissection due to Hyperleptinemia. A) Distribution of TAD patients across BMI categories. B) Aortic diameters measured in TAD patients stratified by BMI (*n* = 16 underweight, *n* = 195 normal weight, *n* = 250 overweight, *n* = 109 obese). C) Hazard ratios for aortic dissection (AD) by BMI category derived from multivariable Cox regression analysis. D) Serum leptin concentrations in healthy controls (*n* = 57) versus TAD patients (*n* = 101; *p* < 0.001). E) Serum leptin concentrations in TAD patients categorized by BMI. F,G) Representative immunohistochemical staining for leptin in human aortic sections (Control: *n* = 26; TAD: *n* = 36; scale bars: 1 mm and 100 µm). H,I) Western blot analysis of leptin expression in aortic tissues from non‐TAD controls and TAD patients (*n* = 18/group). J,K) Immunohistochemical detection of leptin in aortic sections from healthy control and TAD mice (*n* = 16/group; scale bars: 300 and 100 µm). L) Serum leptin concentrations in control versus TAD mice (*n* = 16/group; *p* < 0.001). M) Relative leptin mRNA levels in aortic tissue from control and TAD mice (*n* = 9/group; P = 0.019). N,O) Western blot analysis of leptin expression in aortic tissues from control and TAD mice (*n* = 6/group). Data represent means ± SEM. Statistical analyses were performed using: Student's t‐test (equal variance; Figure N), Welch's t‐test (unequal variance; Figure H,K), multiple t‐tests (Figure E), Mann‐Whitney U test (non‐parametric data; Figure D,M,G,L), or one‐way ANOVA with post‐hoc correction for multiple comparisons (Figure B).

**Figure 2:**
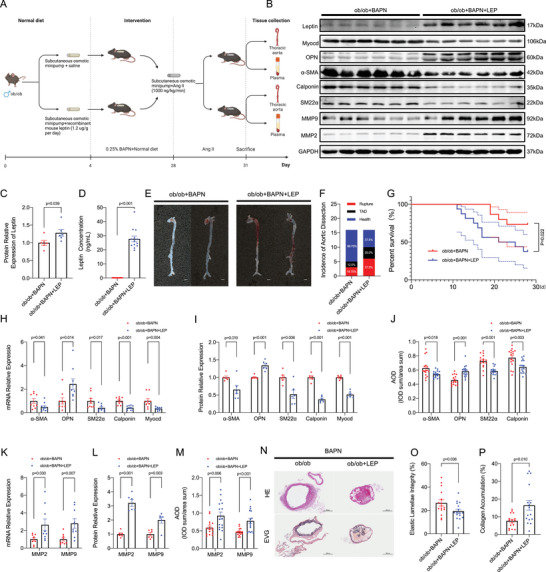
Exogenous Leptin Supplementation Exacerbates Thoracic Aortic Dissection in Leptin‐Deficient Mice. A) Experimental timeline: Four‐week‐old male ob/ob mice received subcutaneous osmotic minipump delivery of recombinant murine leptin (1.2 µg g^−1^ day^−1^) or saline concurrently with 0.25% BAPN administration in drinking water for 28 days. Angiotensin II (Ang II, 1000 ng kg^−1^ min^−1^) was infused via mini‐osmotic pump during the final 3 days prior to sacrifice. B,C) Western blot analysis of leptin expression in aortic tissues from leptin‐treated versus saline‐treated ob/ob mice (*n* = 6/group). D) Serum leptin concentrations (*n* = 13/group). E) Macroscopic aortic morphology (scale bar: 2 mm). F) Incidence of thoracic aortic dissection (TAD). G) Kaplan‐Meier survival analysis compared by log‐rank test (P = 0.022; n = 16/group). H–J) mRNA expression (H), Western blot (I), and immunohistochemical staining (J) of contractile (α‐SMA, SM22α, Calponin, MYOCD) and synthetic (OPN) phenotypic markers in aortic sections. K–M) mRNA expression (K), Western blot (L), and immunohistochemical staining (M) of MMP2/9 in aortic sections. N) Representative hematoxylin and eosin (HE) and elastin Van Gieson (EVG) staining of aortic sections (*n* = 16; scale bars: 300 and 100 µm). O,P) Quantification of elastic lamellae integrity (O) and collagen deposition (P). Data represent means ± SEM. Statistical analyses utilized: Student's t‐test (equal variance; Figure C), Welch's t‐test (unequal variance; Figure O), multiple t‐tests (Figure H–M), and Mann‐Whitney U test (non‐parametric data; Figure D,P).

From 2012 to 2022, our cohort enrolled a total of 1017 patients diagnosed with thoracic aortic dissection (TAD). Notably, 68.93% of these patients were either obese (BMI ≥ 28 kg m^−2^) or overweight (24 ≤ BMI < 28 kg m^−2^), as illustrated in **Figure** **1**A. When comparing aortic diameters across different BMI categories, both obese and overweight patients exhibited significantly larger diameters compared to those in other BMI groups, aligning with the prevalent perception that obesity is prevalent among TAD patients, as depicted in Figure 1B. To validate our findings, we analyzed UK Biobank data encompassing 498 267 participants. Consistently, we observed that 77.37% of patients with acute aortic dissection (AD) were either obese or overweight (Table S1, Supporting Information), corroborating the trends observed in our cohort (Figure 1A; Table S2, Supporting Information). Further multivariate Cox regression analysis revealed a heightened risk of aortic dissection among overweight and obesity individuals, as shown in Figure 1C and Table S3 (Supporting Information). Additionally, ELISA testing (Figure 1D) demonstrated a significant elevation in leptin expression among TAD patients. This increase was particularly pronounced in obese and overweight TAD patients compared to their non‐obese counterparts (Figure 1E). To explore the potential link between leptin and BMI, Mendelian randomization (MR) analysis^[^ ^17^, ^18^, ^19^, ^20^, ^21^ ^]^ was conducted. The results demonstrated a robust association between BMI and the risk of aortic aneurysm (AA) (p = 1.38E‐07). However, when the influence of leptin was accounted for, this correlation was substantially attenuated (p = 0.307), as presented in Figure S1 and Table S4 (Supporting Information). Immunohistochemical staining (Figure 1F and **Figure** **2**G), Western blot analysis (Figure 2H,I) consistently demonstrated enhanced leptin content in aortic tissues of TAD patients compared to healthy controls or those without TAD. Similarly, leptin content was upregulated in the aorta and serum of BAPN‐induced and Ang II‐induced TAD mice, as evidenced by immunohistochemical staining (Figure 1J,K; Figure S2A,B, Supporting Information), ELISA (Figure 1L), qPCR (Figure 1M), and Western blot analysis (Figure 1N,O; Figure S2C,D, Supporting Information). Collectively, these data implicate leptin as a potential contributor to TAD pathogenesis.

### 2.2 Leptin Aggravates Thoracic Aortic Dissection Formation with Promoted VSMCs Contractile‐to‐Synthetic Phenotypic Transitions

To further investigate the role of leptin in thoracic aortic dissection (TAD), we employed leptin‐knockout (ob/ob) mice in a BAPN‐induced TAD model. Male ob/ob mice aged 4 weeks were subjected to subcutaneous leptin or saline administration using an osmotic minipump^[^ ^22^, ^23^ ^]^ (*n* = 16 each) to assess its impact on TAD pathogenesis (Figure 2A). ELISA and Western blot (WB) analysis confirmed a significant elevation of leptin levels in both serum and aortic tissues in comparison to controls, validating the successful administration of leptin (Figure 2B–D). Notably, leptin administration led to a significant decrease in body weight, indicating its systemic effects (Figure S3A, Supporting Information). Mortality rates attributed to aortic dissection and rupture were notably higher in leptin‐treated mice, reaching 62.5%, compared to 31.25% in controls (Figure 2E,G). Similarly, the incidence of aortic dissection was also increased in leptin‐treated mice, with a rate of 52.5% compared to 31.25% in controls (Figure 2E,F). In‐depth molecular analysis using WB, mRNA analysis, and immunohistochemistry revealed that leptin administration led to a decrease in contractile markers (α‐SMA, SM22α, Calponin, MYOCD) and an increase in synthetic markers (OPN) as well as ECM‐degrading enzymes (MMP2/9) in thoracic aorta tissues (Figure 2H–M; Figure S3B–G, Supporting Information). These changes suggest that leptin may promote a phenotypic transition in thoracic aorta tissues, favoring a more synthetic and ECM‐degrading phenotype. To further corroborate our findings, we conducted in vitro studies which demonstrated that these changes were leptin concentration‐dependent, providing further evidence for the direct role of leptin in modulating VSMC phenotype (Figure S4A–E, Supporting Information). Histological analysis of aortic tissues from leptin‐treated mice revealed exacerbated aortic dissection, elastin disruption, and collagen deposition, indicating a more severe pathological phenotype compared to controls (Figure 2N–P; Figure S3H, Supporting Information). Collectively, these findings suggest that leptin may exacerbate TAD in mice through its modulatory effects on VSMC phenotype and ECM homeostasis, providing important insights into the potential role of leptin in the pathogenesis of TAD.

### 2.3 Pharmacological Inhibition of Leptin Protects Against BAPN‐Induced Thoracic Aortic Dissection

**Figure 3:**
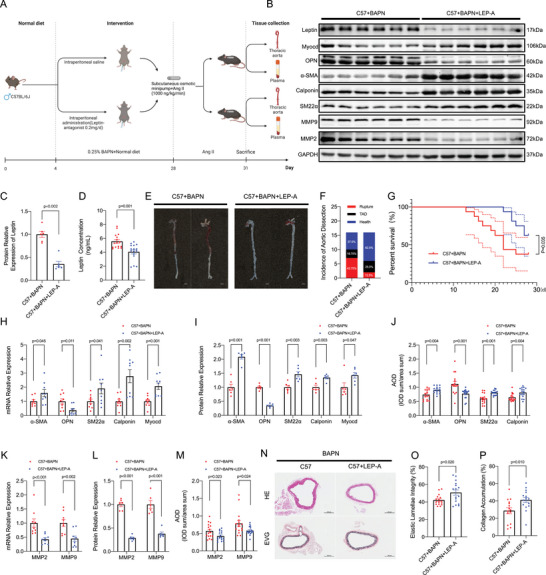
Leptin Antagonist (R128Q/ LEP‐A) Alleviates TAD. A) Experimental timeline. C57BL/6JGpt mice received 0.25% BAPN in drinking water for 28 days. Angiotensin II (Ang II, 1000 ng kg^−1^ min^−1^) was infused via osmotic minipump for the final 3 days. R128Q (LEP‐A; 0.2 mg d^−1^) or saline was administered intraperitoneally daily throughout the modeling period. B,C) Western blot analysis of leptin levels in aortic tissue (*n* = 6/group). D) Serum leptin concentration (*n* = 16/group). E) Representative aortic images (scale bar: 2 mm). F) TAD incidence. G) Survival analysis by Kaplan‐Meier method, compared using log‐rank test (P = 0.035; *n* = 16/group). H–J) mRNA expression (H), Western blot (I), and immunohistochemical staining (J) of contractile (α‐SMA, SM22α, Calponin, MYOCD) and synthetic (OPN) phenotypic markers in aortic sections. K–M) mRNA expression (K), Western blot (L), and immunohistochemical staining (M) of MMP2/9 in aortic sections. N) Representative hematoxylin and eosin (HE) and elastin Van Gieson (EVG)‐stained aortic sections (*n* = 16/group; scale bars: 300 and 100 µm). O,P) Quantification of elastic lamellae integrity (%) (O) and collagen accumulation (%) (P). Data represent mean ± SEM. Statistical analyses: Welch's t‐test (Figure O); Student's t‐test (Figure P); multiple t‐tests (Figure H–M); Mann‐Whitney U test (Figure C,D). AOD: Average optical density.

**Figure 4:**
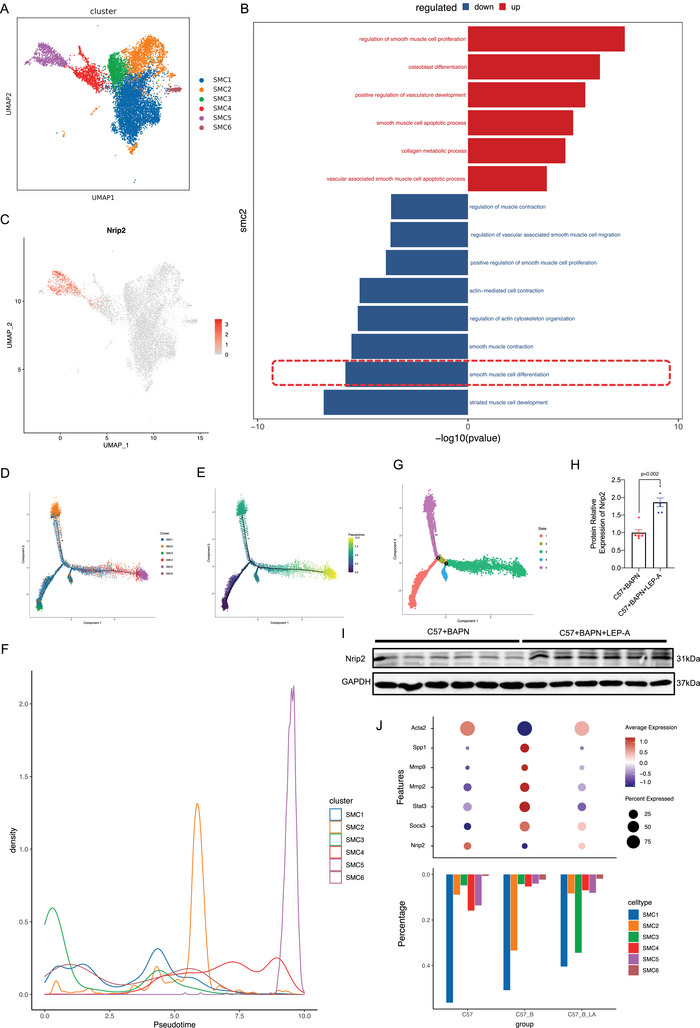
Nrip2^+^ Vascular Smooth Muscle Cells Preserve the Contractile Phenotype During Thoracic Aortic Dissection. A) Uniform Manifold Approximation and Projection (UMAP) visualization of six distinct SMC subclusters identified through single‐cell RNA sequencing. B) Significantly enriched Gene Ontology terms for cluster 2 SMCs. C) Spatial distribution of Nrip2 expression across SMC subclusters overlaid on UMAP coordinates. D,E) Pseudotime trajectory reconstruction of SMCs, with points colored by subcluster identity (D) or progression along pseudotime (E). F) Density distribution of SMC subclusters across pseudotime. G) Pseudotime trajectory segmented by differentiation states. H,I) Western blot analysis quantifying Nrip2 protein expression in aortic tissues from BAPN‐induced TAD mice with or without leptin antagonist (LEP‐A) treatment (*n* = 6/group). J) Upper panel: Dot plot displaying average expression (color intensity) and detection frequency (dot size) for key genes across experimental groups. Lower panel: Proportional distribution of SMC subclusters among treatment groups. Data represent mean ± SEM; The Mann‐Whitney U test was utilized for non‐normally distributed variables (Figure H).

To further assess the therapeutic potential of leptin inhibition, we conducted a study using the leptin antagonist R128Q (LEP‐A), the recombinant human leptin mutant with potent antagonistic efficacy through its ability to competitively bind the leptin receptor (ObRb) with affinity comparable to wild‐type leptin while failing to initiate signal transduction due to impaired receptor dimerization.^[^ ^24^ ^]^ Post‐BAPN administration, mice were treated with daily intraperitoneal R128Q (0.2 mg d^−1^) for a duration of 4 weeks (**Figure** **3**A).^[^ ^25^ ^]^ ELISA and Western blot (WB) analysis were performed to evaluate the effects of R128Q (LEP‐A) treatment on leptin content Our results revealed a significant reduction in leptin expression in both aortic tissues and serum following R128Q (LEP‐A) treatment (Figure 3B–D), indicating effective leptin antagonism. Remarkably, mice receiving R128Q (LEP‐A) (C57+BAPN+LEP‐A) exhibited a lower incidence of TAD (**Figure** **4**E,F) and mortality (Figure 3E,G) compared to control mice (C57+BAPN) (*n* = 16 each). This observation suggests that leptin antagonism may attenuate the progression of TAD. In the context of R128Q (LEP‐A) treatment administered alone, minimal changes were observed in body weight or systolic blood pressure (Figure S5A,B, Supporting Information). Further findings revealed that R128Q (LEP‐A) treatment led to an increase in contractile markers (α‐SMA, SM22α, Calponin, MYOCD) and a decrease in synthetic markers (OPN) as well as ECM‐degrading enzymes (MMP2/9) in thoracic aorta tissues (Figure 3H–M; Figure S5B–G, Supporting Information). These changes suggest that leptin antagonism may restore the contractile phenotype of VSMCs and attenuate ECM degradation, thereby preventing the progression of TAD. Pathological analysis of aortic tissues further corroborated our findings. Mice treated with R128Q (LEP‐A) exhibited rescued elastin fibers, reduced collagen deposition, and prevented aortic wall rupture compared to controls (Figure 3N–P; Figure S5H, Supporting Information). These histological improvements indicate a favorable therapeutic effect of leptin antagonism on aortic tissue integrity. Collectively, our findings underscore the important role of leptin in the progression of TAD and suggest that R128Q (LEP‐A) may serve as a potential therapeutic agent for this condition. By antagonizing leptin's effects, R128Q (LEP‐A) may restore VSMC function and ECM homeostasis, thereby preventing the development and progression of TAD.

### 2.4 Single‐Cell RNA Sequencing Reveals Crucial Role of Nrip2^+^ VSMCs in Leptin‐Mediated Thoracic Aortic Dissection

To gain a deeper understanding of leptin's involvement in the phenotypic transition of VSMCs during TAD, we performed single‐cell RNA sequencing on mouse aortic tissues. Through integration and clustering analysis, we identified six distinct VSMC clusters (Figure 4A). Expression analysis revealed upregulation of genes such as Spp1, Stat3, and Socs3, along with downregulation of Acta2 and Tagln, in the aortas of TAD mice compared to controls. Notably, treatment with the leptin antagonist LEP‐A reversed these trends, suggesting a role for leptin in modulating VSMC phenotype (Figure S6A,B, Supporting Information). Pseudotime analysis further revealed that SMC cluster 2, characterized by high Spp1 expression in TAD mice, exhibited a decrease in Spp1 expression following LEP‐A treatment. GO analysis indicated downregulation of differentiation‐related functions in cluster 2 of TAD mice, which was upregulated by LEP‐A treatment (Figure 4B; Figure S6E, Supporting Information). Additionally, the upregulation of Stat3, and Socs3 in cluster 2 was reversed by LEP‐A treatment, the same trend as Spp1 and Acta1 (Figure S6C,D, Supporting Information). Furthermore, our analysis identified cluster 5, which highly expressed Nrip2, as a distinct subpopulation of VSMCs (Figure 4C). Pseudotime analysis suggested that Nrip2^+^ SMCs and cluster 2 occupy different branches in the phenotypic transition, indicating that Nrip2^+^ SMCs may represent a more mature and differentiated cell population (Figure 4D–G; Figure S7A–C, Supporting Information). Sequencing data also showed an increase in the proportion of Nrip2^+^ VSMCs following LEP‐A treatment, which was further confirmed by in vivo experiments (Figure 4H–J). Additionally, contractile phenotypic markers were upregulated in the LEP‐A‐treated group, highlighting the potential role of leptin antagonism in promoting a contractile VSMC phenotype (Figure 4J). Collectively, these data suggest that Nrip2^+^ VSMCs exhibit contractile properties and may play a critical role in maintaining vascular homeostasis.

### 2.5 Leptin Facilitates the Phenotypic Transition of Smooth Muscle Cells from Contractile to Synthetic via Nrip2‐Dependent Mechanisms

**Figure 5:**
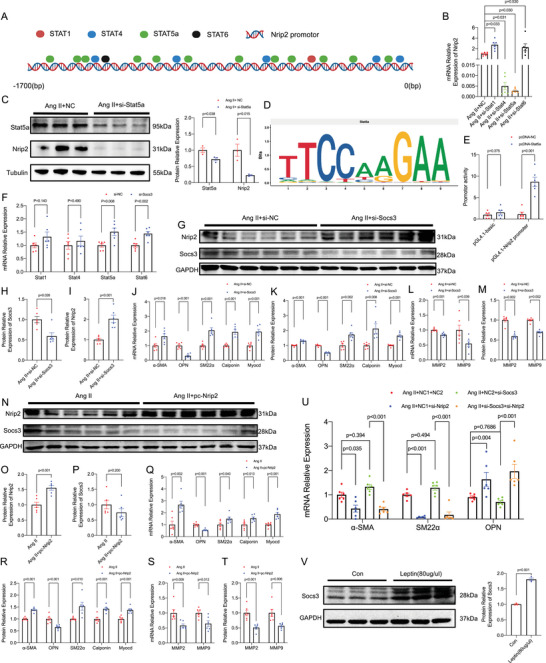
Nrip2 Governs Phenotypic Transitions via the Socs3‐Stat5a Pathway. A) Transcription factor binding sites on the Nrip2 promoter predicted using PROMO. B) Nrip2 mRNA levels in VSMCs following transcription factor siRNA knockdown (*n* = 6). C) Representative Western blots and quantification of Stat5a and Nrip2 protein in VSMCs transfected with Stat5a siRNA (*n* = 3). D) Stat5a binding motif identified by JASPAR. E) Luciferase reporter assays showing Nrip2 promoter activity normalized to Renilla luciferase (*n* = 6). F) mRNA levels of transcription factors after Socs3 siRNA treatment (*n* = 6). G–I) Western blots and quantification of Socs3 (H) and Nrip2 (I) in Socs3 siRNA‐treated VSMCs (*n* = 6). J–M) mRNA levels (J), Western blots, and quantification (K) of contractile/synthetic markers, and mRNA (L)/protein (M) levels of MMP2/9 in Socs3 siRNA‐stimulated SMCs (*n* = 6). N–P) Western blots and quantification of Nrip2 (O) and Socs3 (P) in Nrip2 plasmid‐transfected VSMCs (*n* = 6). Q–T) mRNA levels (Q), Western blots, and quantification (R) of phenotypic markers, and mRNA (S)/protein (T) levels of MMP2/9 in Nrip2 plasmid‐stimulated SMCs (*n* = 6). U) Phenotypic marker mRNA in VSMCs co‐transfected with Socs3 and Nrip2 siRNAs (*n* = 6). V) Western blots and quantification of Socs3 in leptin‐treated (80 µg mL^−1^) VSMCs (*n* = 3). Data represent mean ± SEM. Statistical analyses: One‐way repeated‐measures ANOVA (Figure B); Two‐way repeated‐measures ANOVA (Figure U); Student's t‐test (Figure O,P,V); Welch's t‐test (Figure I); Multiple t‐tests (Figure C,E,F,J–M,Q–T); Mann‐Whitney U test (Figure H); One‐way ANOVA (Figure B) or Two‐way ANOVA (Figure U) with post‐hoc correction.

To investigate the regulatory mechanisms of Nrip2, we employed the PROMO program to predict transcription factor binding sites on the Nrip2 promoter. Restricting the dissimilarity margin to 3%, we identified multiple Stat family binding sites, including Stat1, Stat4, Stat5a, and Stat6 (**Figure** **5**A). siRNA‐mediated knockdown of Stat5a, rather than Stat1, Stat4, or Stat6, significantly reduced Nrip2 expression in VSMCs (Figure 5B,C; Figure S8A–D, Supporting Information), suggesting Stat5a as a key upstream transcriptional factor for Nrip2. Using JASPAR, we predicted the binding motif sequence of Stat5a (Figure 5D). Dual‐luciferase reporter assays confirmed the activation of the Nrip2 promoter by overexpressing Stat5a (Figure 5E). Since the Stat family is negatively regulated by Socs3, we observed an increase in Stat5a and Stat6 expression after Socs3 knockdown (Figure 5F). These findings provide insights into the transcriptional regulation of Nrip2 and its potential role in VSMC phenotypic modulation.

To further validate the regulatory roles of Socs3 and Nrip2 in TAD progression, murine primary aortic VSMCs were transfected with Socs3 siRNA or Nrip2 plasmids (Figure S8E,F, Supporting Information). Knockdown of Socs3 in VSMCs upregulated Nrip2 and contractile markers while downregulating OPN and MMP2/9 expression (Figure 5G–M; Figure S8G,H, Supporting Information). Conversely, transfection with Nrip2 plasmids potentiated the contractile phenotype of VSMCs and suppressed OPN and MMP2/9 expression, without affecting Socs3 levels (Figure 5N–T; Figure S8I,J, Supporting Information). When both Socs3 and Nrip2 were knocked down simultaneously in VSMCs, phenotypic markers exhibited similar trends to those observed in cells treated with si‐Nrip2 alone, but contrasted with the pattern in cells treated with si‐Socs3 alone (Figure 5U; Figure S8K–M, Supporting Information). Furthermore, our investigation revealed a significant upregulation of Socs3 expression in VSMCs upon leptin intervention (Figure 5V). These findings implicate that Socs3 regulates phenotypic transitions, likely through its downstream target Nrip2.

### 2.6 Nrip2 Deficiency Triggered by Periaortic AAV9‐shNrip2 Application Intensifies BAPN‐Induced VSMC Phenotypic Transition Toward Synthetic Phenotype and Elevates TAD Incidence

**Figure 6:**
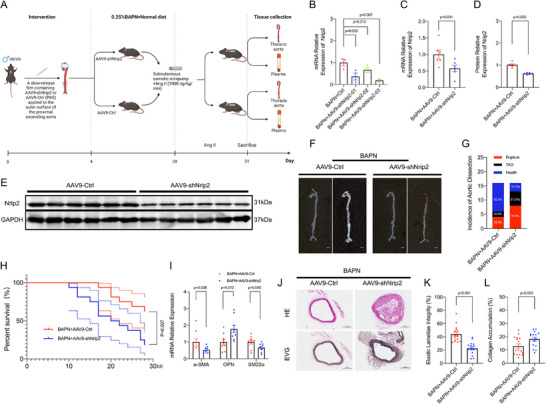
Nrip2 Knockdown Exacerbates BAPN‐Induced VSMC Phenotypic Transition and Thoracic Aortic Dissection in Leptin‐Deficient (ob/ob) Mice. A) Experimental timeline: Leptin‐knockout (ob/ob) mice received 0.25% BAPN in drinking water for 28 days. Concurrently, slow‐release films containing AAV9‐shNrip2 or AAV9‐Ctrl were applied peri‐aortically to the proximal ascending aorta throughout this period. From days 28 to 31, subcutaneous osmotic minipumps delivered angiotensin II (Ang II, 1000 ng kg^−1^ min^−1^). B) Nrip2 mRNA levels in aortic tissues treated with distinct shRNAs (*n* = 6). C) Quantitative analysis of Nrip2 mRNA expression in aortic sections (*n* = 6/group). D,E) Western blot detection of Nrip2 protein in aortas from ob/ob mice receiving periaortic application of slow‐release AAV9‐shNrip2 film versus control (*n* = 6/group). F) Representative aortic photomicrographs (scale bar: 2 mm). G) Incidence of thoracic aortic dissection (TAD). H) Kaplan‐Meier survival analysis with log‐rank test (P = 0.007; *n* = 16/group). I) mRNA quantification of VSMC phenotypic markers in aortic tissues. J) Histological sections stained with hematoxylin‐eosin (HE) and elastin Van Gieson (EVG) (*n* = 16/group; scale bars: 300 and 100 µm). K,L) Quantitative assessment of elastic lamellae integrity (K) and collagen deposition (L). Data represent mean ± SEM. Statistical analyses: Student's t‐test (normally distributed data: Figure C,L), Welch's t‐test (unequal variance: Figure D), multiple t‐tests (I), Mann‐Whitney U test (nonparametric data: Figure K). One‐way ANOVA with post‐hoc correction was applied for multi‐group comparisons (Figure B).

To further elucidate the role of Nrip2 in disease progression, we employed a strategy involving the application of a slow‐release film containing AAV9‐shNrip2 to the proximal ascending aorta of leptin‐knockout (ob/ob) mice. This approach aimed to knock down Nrip2 expression in the aorta tissues (**Figure** **6**A–E). Our findings revealed that mice in the AAV9‐shNrip2 group exhibited significantly higher mortality rates due to aortic dissection and rupture, at 81.25% (n = 13), compared to the AAV9‐Ctrl group, where the rate was 37.5% (*n* = 6) (Figure 6F,H). Similarly, the incidence of aortic dissection was also higher in the AAV9‐shNrip2 group, accounting for 81.25% of cases, compared to 37.5% in the AAV9‐Ctrl group (Figure 6F,G). Moreover, the knockdown of Nrip2 in mouse aortas by AAV9‐shNrip2 resulted in reduced expression of both Nrip2 and contractile proteins, accompanied by an upregulation of OPN in aortic tissues (Figure 6E,I). Histological examinations further revealed augmented collagen deposition, elastin disruption, and dissection formation in the intervention group compared to the controls (Figure 6J–L; *n* = 16 per group). Taken together, these findings suggest that knockdown of Nrip2 in VSMCs may potentially increase the risk of TAD in mice.

### 2.7 Nrip2 Overexpression by Periaortic AAV9‐Nrip2 Application Alleviates TAD Progression and Reverses BAPN‐Induced VSMC Phenotypic Transition in The Aorta

**Figure 7:**
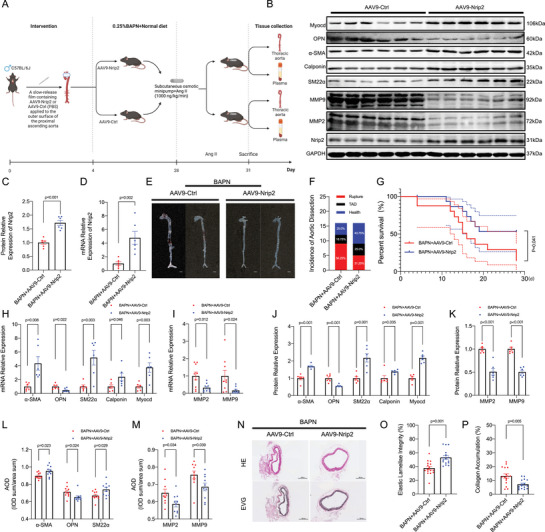
Overexpression of Nrip2 in VSMCs Decreases the Incidence of TAD via Maintaining VSMC Contractile Phenotype. A) Experimental timeline: C57BL/6J mice received 0.25% BAPN in drinking water for 28 days. Throughout this period, slow‐release films containing AAV9‐Nrip2 or AAV9‐Ctrl were applied peri‐aortically to the proximal ascending aorta. From days 28 to 31, subcutaneous osmotic minipumps delivered angiotensin II (Ang II, 1000 ng kg^−1^ min^−1^). B,C) Western blot analysis of NRIP2 protein expression in aortic tissues (*n* = 6/group). D) Quantification of Nrip2 mRNA levels in aortic sections (*n* = 6). E) Representative aortic macrographs (scale bar: 2 mm). F) Thoracic aortic dissection (TAD) incidence. G) Survival analysis by Kaplan‐Meier method with log‐rank test (P = 0.041; *n* = 16/group). H,I) mRNA abundance of VSMC phenotypic markers (H) and MMP2/9 (I) in aortic tissues. J,K) Western blot quantification of contractile markers (J) and MMP2/9 (K). L,M) Immunohistochemical analysis of phenotypic markers (L) and MMP2/9 (M) (*n* = 10/group). N) Representative aortic sections stained with hematoxylin‐eosin (H&E) and elastin van Gieson (EVG) (*n* = 16/group; scale bars: 300 and 100 µm). O,P) Quantitative analysis of elastic lamellae integrity (%) (O) and collagen accumulation (%) (P). Data represent mean ± SEM. Statistical analyses: Student's t‐test (Figure 7C), Welch's t‐test (Figure 7D,P), multiple t‐tests (Figure 7H–M), and Mann‐Whitney U test (Figure 7O).

To investigate the functional significance of Nrip2 in disease progression, we administered a slow‐release film containing AAV9‐Nrip2 and sm22a‐promoter to the proximal ascending aorta of C57BL/6J mice, aiming to enhance Nrip2 expression specifically in VSMCs in the aorta tissues (**Figure** **7**A–D; Figure S9A,B, Supporting Information). Our findings indicate that while the control group (AAV9‐Ctrl, *n* = 12) exhibited a mortality rate of 75.0% due to aortic dissection and rupture, the mortality rate in the intervention group overexpressing Nrip2 (AAV9‐Nrip2, *n* = 9) was slightly lower at 56.25% (Figure 7E,G). However, the difference in the incidence of aortic dissection between the two groups was minimal, with 75.00% in the control group and 56.25% in the intervention group (Figure 7E,F). Notably, mice overexpressing Nrip2 through AAV9‐Nrip2 treatment exhibited reduced expression of synthetic proteins and MMP2/9, coupled with upregulated levels of Nrip2 and contractile proteins in their aortic tissues (Figure 7B,H–M; Figure S9A–H, Supporting Information). Furthermore, histological analysis revealed attenuated dissection formation, collagen deposition, and elastin disruption in the intervention group compared to the control group (Figure 7N–P; Figure S9H, Supporting Information; *n* = 16 per group). Taken together, these findings suggest that an augmentation of Nrip2^+^ VSMCs expression may potentially decrease the incidence of thoracic aortic dissection (TAD) in mice.

### 2.8 Enhanced Nrip2 Expression in VSMCs Boosts Mitochondrial Energy Metabolism, thereby Maintaining VSMCs in A Contractile State

**Figure 8:**
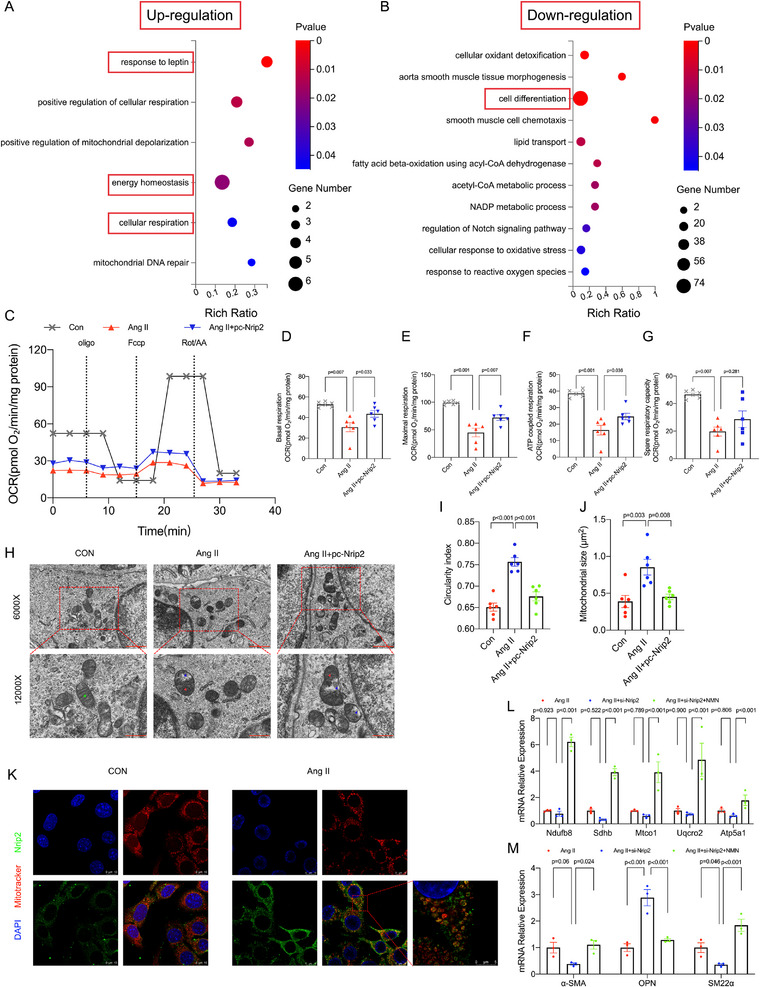
An Elevated Population of Nrip2^+^ VSMCs Maintains VSMCs in a Contractile Phenotype by Improving Mitochondrial Energy Metabolism. A,B) Gene Ontology (GO) enrichment analysis (biological process) of upregulated (A) and downregulated (B) pathways from VSMC transcriptomics following Nrip2 plasmid transfection versus control. C) Representative traces and D–G) quantitative analysis of mitochondrial respiration parameters in Nrip2‐transfected VSMCs (*n* = 6): basal OCR (D), maximal OCR (E), ATP‐coupled OCR (F), and spare respiratory capacity OCR (G). H–J) Representative images of mitochondrial ultrastructure using TEM in VSMCs (H, green ▲: The density of mitochondria was normal without fragmentation or cristae disorganization.red▲: Mitochondria with cristae disorganization. blue▲: Mitochondrial swelling rupture.), and quantification of circularity index(I) and mitochondrial size(J) based on TEM images (*n* = 6 per group). K) Immunofluorescence showing Nrip2 (green) co‐localization with mitochondria (red) following 100 µm Ang II stimulation (24 h). DAPI stains nuclei. Scale bars: 10 µm (overview), 5 µm (inset). L) mRNA levels of mitochondrial complex genes. M) mRNA abundance of VSMC phenotypic markers after NMN treatment in Nrip2‐insufficient VSMCs. Data represent mean ± SEM. Statistics: One‐way ANOVA (Figure D–G,I,J); Two‐way ANOVA (Figure L,M) with post‐hoc correction.

To elucidate the downstream processes modulated by Nrip2 in VSMCs, transcriptome sequencing was conducted on VSMCs treated with Nrip2 plasmids (**Figure** **8**A). GO enrichment analysis unveiled significant perturbations in cellular responses to leptin and cell differentiation, with a particular emphasis on mitochondrial and cellular respiration‐related pathways (Figure 8B). Functional validation using mitochondrial respiratory assays demonstrated that Nrip2 overexpression elevated basal, ATP‐coupled, and maximal oxygen consumption rates (OCR), indicating augmented mitochondrial energy metabolism (Figure 8C–G). Transmission electron microscopy further showed that Nrip2 overexpression mitigated Ang II‐induced mitochondrial abnormalities, including disorganization, fragmentation, and cristae disruption, while restoring normal mitochondrial density, ultrastructure, circularity, and size (Figure 8H–J). Immunofluorescence confirmed Nrip2 co‐localization with mitochondria following Ang II stimulation (Figure 8K). Crucially, suppression of mitochondrial complex activities observed under Nrip2 deficiency was rescued by NMN supplementation (Figure 8L). This intervention also reversed the shift from a contractile to synthetic phenotype in Nrip2‐deficient VSMCs (Figure 8M). Collectively, these findings indicate that Nrip2 sustains the VSMC contractile state by enhancing mitochondrial bioenergetics and structural integrity, while NMN administration compensates for Nrip2 deficiency, suggesting therapeutic potential for mitochondrial‐targeted strategies.

## 3 Discussion

Our findings revealed that leptin promotes the decrease of Nrip2^+^ VSMCs and impairs energy metabolism through the Socs3‐Stat5a‐Nrip2 signalling pathway, offering a novel therapeutic approach and target for preventing TAD. Enhanced Nrip2 expression effectively mitigates leptin‐induced ECM degradation and VSMC phenotypic transitions during TAD progression.

Epidemiological analyses, notably from the UK Biobank, consistently identify obesity as a significant risk factor for thoracic aortic dissection (TAD), a correlation supported by extensive clinical observations. Although regional BMI criteria differ slightly (e.g., China defines overweight as BMI ≥ 24 kg m^−^^2^ vs WHO's ≥ 25 kg m^−^^2^), both standards consistently identified obesity as a significant TAD risk factor, underscoring the robustness of this association. This association is partly mediated through hypertension, a frequent comorbidity in both obesity and TAD, which chronically contributes to arterial wall degeneration.^[^ ^27^ ^]^ This degeneration involves smooth muscle cell phenotypic transition, atherosclerosis, and progressive degradation of elastic and collagen fibers, ultimately weakening the vessel wall.

Obesity is frequently characterized by hyperleptinemia and leptin resistance,^[^ ^28^ ^]^ leading to elevated circulating leptin levels, which is a known risk factor for cardiovascular diseases.^[^ ^29^, ^30^, ^31^, ^32^ ^]^ This resistance results in decreased leptin effectiveness, necessitating higher levels of leptin secretion to maintain metabolic functions. However, hyperleptinemia can significantly increase the risk of thoracic aortic dissection due to its adverse vascular effects.^[^ ^33^, ^34^ ^]^ While our single‐center data confirm increased leptin levels in overweight/obese TAD patients and suggest a role for leptin in TAD pathogenesis via adverse vascular effects, conflicting evidence exists in a study finding no independent association between leptin and abdominal aortic aneurysms (AAAs).^[^ ^35^ ^]^ This discrepancy may be attributed to several key distinctions. Anatomically, TAD and AAA involve divergent embryological origins (neural crest vs mesodermal VSMCs), inflammatory milieus, and mechanical stresses^[^ ^36^ ^]^; crucially, our single‐cell RNA sequencing identified a thoracic‐enriched Nrip2^+^ VSMC subpopulation mediating leptin's metabolic effects, which may not operate equivalently in abdominal segments. Mechanistic differences are further underscored by TAD's rapid elastolysis under high shear stress versus AAA's atherogenic progression. Methodologically, their analysis used banked sera from elderly males (mean age 72 years), where comorbidities and medications (e.g., statins) could confound leptin signaling, whereas our samples were collected acutely (<48 h post‐symptom onset) prior to hemodilution or intervention, capturing bioactive leptin dynamics. Additionally, prior studies did not differentiate free versus receptor‐bound leptin fractions, whereas soluble leptin receptor (sOb‐R) levels—reduced in obesity—modulate bioactivity and were unassessed.^[^ ^37^ ^]^ Importantly, our work delineates a previously unrecognized Socs3‐Stat5a‐Nrip2‐mitochondrial axis driving VSMC phenotypic transitions, a pathway unexplored in earlier AAA research. Thus, while leptin may not universally drive all aortic pathologies, its context‐dependent impact on thoracic‐specific VSMC subpopulations and bioenergetic failure substantiates its role in TAD pathogenesis.

Our study reveals that leptin plays a pivotal role in promoting the occurrence and development of thoracic aortic dissection (TAD). Based on these findings, we have successfully designed a leptin mutant protein, designated as LEP‐A/R128Q,^[^ ^26^, ^38^ ^]^ which functions as a leptin antagonist. This mutant protein effectively disrupts leptin's receptor binding, thus inhibiting its subsequent biological activity. Importantly, LEP‐A/R128Q is non‐chemically toxic with metabolic and physiological safety, as evidenced by the absence of adverse physiological, histological, or metabolic alterations at therapeutic doses.^[^ ^39^, ^40^ ^]^ Our preliminary results indicate that LEP‐A mitigates phenotypic transitions in aortic vascular smooth muscle cells, ECM degradation, and the progression of TAD. Notably, this effect is accompanied by a significant reduction in leptin levels within the aortic tissues and serum of mice. These encouraging outcomes underscore the therapeutic potential of leptin antagonists in treating TAD and related cardiovascular conditions. However, it is crucial to emphasize that further studies are imperative to translate these preclinical findings into clinical practice. We are committed to exploring the mechanisms underlying LEP‐A's effects and assessing its long‐term safety and efficacy in larger animal models. Such efforts are essential for validating the therapeutic potential of leptin antagonists and potentially opening new avenues for the treatment of TAD and associated cardiovascular disorders.

To enhance our comprehension of leptin's function and mechanism in TAD, we conducted single‐cell level studies in mice treated with LEP‐A/BAPN. Our findings illustrated that the process of phenotypic transition was significantly changed in VSMCs under TAD, in agreement with previous studies.^[^ ^41^ ^]^ We singled one distinct VSMC subpopulation (Nrip2^+^ SMC) that was at the end of the pseudotime axis. Combined with their functional properties, we presumed Nrip2^+^ VSMC as subpopulations with contractile characteristics. More importantly, Nrip2 plays an important role in maintaining the contractile phenotype of VSMCs. Nrip2 was initially described as a suppressor of the nuclear receptor in the mouse brain and as a Wnt pathway interactor in regulating colorectal cancer‐initiating cell renewal.^[^ ^42^ ^]^ However, investigation into Nrip2 in cardiovascular diseases is limited. Zhao et al. inferred that Nrip2 probably participates in VSMC regulation.^[^ ^43^ ^]^ For further investigation of the role of Nrip2 in TAD, overexpression with AAV9‐Nrip2 was used and found to mitigate TAD incidence in vivo and prevent the transition from a pro‐contractile to a pro‐synthetic phenotype. To explore the possible mechanisms that regulate Nrip2, the prediction tool (PROMO) indicates that Stat5a is the direct transcript factor of Nrip2. Stat5, which has two paralogs stat5a and stat5b, are proteins involved in a variety of critical cellular functions and pathways. It has been reported that Stat5a was expressed in the media of AAA and non‐aneurysmal abdominal aorta.^[^ ^44^ ^]^ In addition, Stat5a silencing could cause VSMC hypertrophy under external stimuli like hyopoxia.^[^ ^45^ ^]^ We speculate that Stat5a may have different effects on VSMC under other external stimuli, and have verified that Stat5a could be inhibited by Socs3 siRNA and then induced lower expression of Nrip2 in VSMCs. Socs3 is a suppressor of cytokine signaling involved in the regulation of leptin signaling and the Stat family.^[^ ^46^ ^]^ Attenuation of Socs3 expression in most diseases can lead to beneficial metabolic effects.^[^ ^47^ ^]^

Mitochondrial dynamics is a pivotal factor regulating phenotypic transitions in vascular smooth muscle cells (VSMCs). Salabei and Hill demonstrated that PDGF‐BB prompts VSMC dedifferentiation into a synthetic phenotype through excessive mitochondrial fission.^[^ ^48^ ^]^ Mitochondrial morphology, whether fragmented or elongated, is intricately linked to cardiovascular diseases like atherosclerosis and myocardial infarction.^[^ ^49^ ^]^ Cooper et al. observed upregulated DRP1 and mitochondrial fission in mouse abdominal aortic aneurysms, associated with mitochondrial dysfunction and decreased VSMC contractility.^[^ ^50^ ^]^ In our study, transcriptomic analysis of vascular smooth muscle cells (VSMCs) treated with Nrip2 plasmids revealed that Nrip2 primarily regulates energy homeostasis within these cells. TEM analysis revealed that Ang II‐induced mitochondrial disorders, including abnormal density and fragmentation, were alleviated by Nrip2 overexpression. Immunofluorescence indicates Nrip2 colocalized with mitochondria in large amounts after treatment with Angiotensin II. This makes us more convinced that Nrip2 alleviates the damage of Angiotensin II stimulation to smooth muscle cells through mitochondria. Furthermore, NMN treatment upregulated the contractile phenotype in Nrip2‐insufficient VSMCs, further supporting the role of the Nrip2‐mitochondrial axis in regulating VSMC phenotype. Specifically, mitochondrial respiratory tests demonstrated a significant increase in basal, ATP‐coupled, and maximal respiration oxygen consumption rates (OCRs) upon overexpression of Nrip2. This observation indicates that Nrip2 plays a crucial role in enhancing mitochondrial energy metabolism in VSMCs. We acknowledge that the specific mechanisms downstream of mitochondria require further investigation, but given that ATP production is essential for maintaining contractile function in VSMCs, these findings suggest that the availability of ATP may be a limiting factor for contractile function. Therefore, maintaining normal mitochondrial dynamics and function through the regulation of Nrip2 appears to be essential for modulating VSMC contractility and vascular wall tension, thereby maintaining vascular health and function.

In patients with thoracic aortic dissection (TAD) and mice, serum leptin levels were elevated, suggesting its potential as a pre‐hospital diagnostic marker to distinguish TAD from healthy controls. Abnormal elevations in leptin appear to act through the Nrip2 axis, leading to disrupted energy metabolism and promoting the transition of VSMCs from a contractile to a synthetic phenotype, thereby contributing to TAD development. Our observations indicate that leptin inhibition can mitigate the progression of TAD. Therefore, targeting leptin‐related pathways, such as reducing serum leptin levels or mitigating Nrip2‐induced mitochondrial dysfunction, may present viable therapeutic strategies for managing TAD.

This study has certain limitations. First, our observational analysis was constrained by confounding variables such as food intake, exercise patterns in mice, and patient medical history. Second, there remains a need for further exploration to clarify the intricate relationship between phenotypic transitions and mitochondrial energy homeostasis. Moreover, while the protective role of LEP‐A in reducing TAD incidence has been demonstrated in mice, additional research is necessary for clinical translation. Future studies should address these limitations to enhance our understanding of TAD pathogenesis.

In summary, we elucidated the pathophysiological role of leptin dysregulation in VSMC phenotypic transition and TAD progression. Mechanistically, aberrant leptin upregulation triggers abnormal energy metabolism through the Nrip2 pathway, thereby promoting phenotypic transitions and TAD development. Both Nrip2 overexpression in the aorta and leptin antagonism can attenuate TAD.

## 4 Experimental Section

### Ethics Approval

The procedures in this study were performed following the Declaration of Helsinki and approved by the Medical Ethics Committee of Fujian Medical University Union Hospital (Approval number: 2019–36). Informed consent from individual patients was not required for the retrospective studies. All subjects provided written informed consent for the study involving aortic tissues and blood samples. All animal experiments were conducted in compliance with the National Institutes of Health Guidelines on the Care and Use of Laboratory Animals (National Institutes of Health Publication, 8th edition, 2011) and approved by the Animal Ethics Committee of Fujian Medical University Union Hospital (Approval number: 2022‐Y‐0763).

### Patient Studies

Patients with TAD hospitalized in the Department of Cardiac Surgery, Fujian Medical University Union Hospital in China from January 2012 to December 2022 were included. Inclusion criteria were as follows: 1) TAD was clinically diagnosed and confirmed using CT angiography or B‐ultrasound; 2) chest pain and other symptoms occurred within 14 days of diagnosis; 3) patients were aged 18–75 years; and 4) emergency surgery was performed within 48 h after admission. BMI (kg m^−2^) was categorized by the criteria of the National Health Commission of the People's Republic of China: normal weight: 18.5 ≤ BMI <24 kg m^−2^, overweight: 24 ≤ BMI <28 kg m^−2^, obesity: BMI ≥ 28 kg m^−2,^ underweight: BMI <18.5 kg m^−2^. The choice, rather than the standard used for UKB, is scientifically justified by well‐documented ethnic differences in body composition and adiposity‐related health risks between Asian and Western populations. Specifically, individuals of Asian descent exhibit higher body fat percentage and central adiposity at lower BMI values compared to Caucasians, leading to an elevated risk of cardiometabolic complications at comparatively lower BMI thresholds.

### UK Biobank (UKB) Epidemiology Study

Data from the UK Biobank, which prospectively followed up with 498 267 participants were analyzed. Multivariable Cox regression, which further adjusted for age, sex, ethnic background, Townsend deprivation index, physical activity, smoking, alcohol intake, and comorbidities, was used to estimate hazard ratios and 95% CIs for the association of BMI with aortic aneurysm and dissection incidence in the UK Biobank. BMI was categorized as follows: underweight (<18.5 kg m^−2^), normal weight (18.5≤ BMI <24.9 kg m^−2^), overweight (25.0≤ BMI <29.9 kg m^−2^), obesity (BMI ≥30.0 kg m^−2^).^[^ ^17^ ^]^ Statistical analyses were performed using SAS 9.4 or R 4.1.2, and *p* < 0.05 was considered statistically significant.

### Mendelian Randomization (MR) Analysis

Publicly available summary‐level genome‐wide association study (GWAS) data were used to perform two‐sample MR.^[^ ^18^ ^]^ GWAS summary‐level data of BMI were obtained from the Genetic Investigation of ANthropometric Traits (GIANT) consortium, GWAS data for leptin from an exome‐based analysis of up to 57 232 individuals, and GWAS data for AD and AA from the FinnGen Project.^[^ ^19^, ^20^, ^21^ ^]^ Univariate MR analysis was performed to examine the causal relationship between BMI and AA or AD risk. Multivariate MR analysis, including leptin as a covariate, was conducted to assess its role in the relationship between BMI and AA/AD. Single‐nucleotide polymorphisms (SNPs) significantly related to exposure (*p* < 5 × 10^−8^) and without linkage disequilibrium (r^2^ < 0.001 within a 10 000‐kb window) were selected as instrumental variables. Estimates were obtained using inverse‐variance weighted MR (IVW‐MR). Statistical analyses were performed using R 4.1.2, and *p*‐values of < 0.05 were considered statistically significant.

### Human Aorta Samples

Clinical aortic tissues and blood samples were obtained from TAD patients after aortic replacement in Fujian Medical University Union Hospital. TAD was diagnosed using clinical history, physical examination, and imaging. Normal thoracic aorta tissues were obtained from heart transplant organ donors. None of these donors had cardiovascular disease; therefore, their vessels could be used as a reference.

### Mouse Model of BAPN‐Induced TAD

Mice (3 weeks old) were provided by Gempharmatech Co., Ltd (SCXK(SU)2018‐0008). The animals were randomly divided into two groups according to the random number table method. A 12:12‐h light‐dark cycle at 20–26 °C with 40–70% relative humidity was maintained. Mice had unlimited access to food and water. They were weighed and underwent quick blood glucose tests every 4 days on an empty stomach. All animals included were male, owing to their low sex hormone variations and high TAD incidence. This design minimized the confounding effects of cyclic hormonal fluctuations in females and aligned with clinical data indicating male predominance in TAD. Future studies are needed to evaluate sex‐specific responses. All mortality events reported in the study were specifically attributed to aortic dissection or rupture in the experimental cohorts. This was systematically documented during daily monitoring, where deaths were immediately assessed via necropsy to confirm aortic‐related pathology (e.g., hemothorax, aortic rupture, or visible dissection). Non‐aortic‐related deaths (e.g., unrelated infections or procedural complications) were excluded from analysis.

Several TAD models were established: i) C57B/6JGpt mice were administered 0.25% 3‐aminopropionitrile fumarate [BAPN, (A0408, TCI, Japan)] in drinking water for 28 days and infused with angiotensin II (Ang II, 1000 ng kg^−1^ min^−1^) with mini‐osmotic pumps (Model 1003D, Alzet) for 3 days to induce TAD^[^ ^22^, ^23^ ^]^; ii) leptin‐knockout mice (ob/ob mice) were administered 0.25% BAPN in water and 1.2 µg g^−1^ recombinant murine leptin or saline via a subcutaneous osmotic minipump for 28 days and infused with angiotensin II (Ang II, 1000 ng kg^−1^ min^−1^) with mini‐osmotic pumps (Model 1003D, Alzet) for 3 days to induce TAD; iii) C57B/6JGpt mice were administered 0.25% BAPN in water and injected with leptin antagonist (R128Q, 100 µg mice^−1^ twice daily)^[^ ^24^ ^]^ for 28 days and infused with angiotensin II (Ang II, 1000 ng kg^−1^ min^−1^) with mini‐osmotic pumps (Model 1003D, Alzet) for 3 days to induce TAD; and iv) C57B/6JGpt mice were administered 0.25% BAPN in water and treated with AAV9‐SM22α‐3xflag‐m‐Nrip2‐Cherry (1.8 × 10^12^ µg mL) by applying gel at the thoracic aorta for 28 days and infused with angiotensin II (Ang II, 1000 ng kg^−1^ min^−1^) with mini‐osmotic pumps (Model 1003D, Alzet) for 3 days to induce TAD. After 3 days, animals were administered an intraperitoneal injection of 2% pentobarbital sodium (100 mg kg^−1^); vital signs were rechecked 10 min after drug administration. Their aortas were then harvested and fixed in 4% paraformaldehyde or stored at −80 °C for subsequent experiments.

### Cell Isolation, Culture, and Treatment

Mouse aortic smooth muscle cells (MASMCs) were isolated from the mouse thoracic aortas. Mouse thoracic aortic tissues were isolated and rinsed in cold PBS. The endothelium and adventitia were removed, and tissues were cut into 1–2‐mm pieces and treated with 0.25% collagenase type II and 0.5% elastase type II at 37 °C for 1 h. MASMCs were maintained in DMEM supplemented with 10% foetal bovine serum and 1% penicillin/streptomycin. Cells from passages 2–4 were harvested for further analysis. Several cell models were used: i) treated with different concentrations of leptin for 48 h; ii) treated with both 100 nmol L^−1^ Ang II and 20 µm siRNA‐Socs3 or 1 µg µL^−1^ pcDNA3.1(+)‐mouse Nrip2‐3xflag (enzyme cutting site: NheI (GCTAGC)‐EcoRI (GAATTC); NM_021717.3) for 48 h. iii) treated with both 100 nmol L^−1^ Ang II and 20 µm Stats (including Stat1, Stat4, Stat5a, and Stat6) siRNA for 48 h; iv) treated with 1 mm NMN (β‐nicotinamide mononucleotide, HY‐F0004) for 48 h. The quality control verification results of the cell intervention can be found in Figures S4, S8, S10 (Supporting Information).

### Single‐Cell Pseudotime Trajectory Analysis: Monocle2

The cell differentiation trajectory of monocyte subtypes was reconstructed with Monocle2 v 2.10.0.^[^ ^25^ ^]^ For constructing the trajectory, the top 2000 highly variable genes were selected by Seurat(v3.1.2) FindVariableFeatures, and dimension‐reduction was performed by DDRTree. The trajectory was visualized by the plot_cell_trajectory function in Monocle2.

### Nrip2 Knockdown In Vivo

To knockdown Nrip2 in mouse aortas, an adeno‐associated virus 9 (AAV9)‐Mir30‐M‐Nrip2 construct (AAV9‐shNrip2) was developed (Figure S9A‐(a–c), Supporting Information). A control vector, AAV‐SM22a‐MCS‐P2A‐mCherry (AAV9‐Ctrl), was also utilized. The AAV9 virus, mixed with hydrogel, was applied peri‐vascularly for one week prior to the administration of 0.25% BAPN in water (Figure 6A).

### Nrip2 Overexpression In Vivo

To overexpress Nrip2 in mouse aortas, adeno‐associated virus 9 (AAV9)‐sm22a‐m‐Nrip2‐3xflag‐mcherry (AAV9‐Nrip2) was constructed (Figure S9A‐(d), Supporting Information). AAV9‐sm22a‐mcherry (AAV9‐Ctrl) was used as the control vector. The AAV9 virus mixed with hydrogel was applied peri‐vascular for 1 week before adding 0.25% BAPN in water (Figure 7A).

### Dual‐Luciferase Reporter Assays

To measure Nrip2 promoter activity, Nrip2 promoter firefly luciferase reporter plasmid pGL3‐Nrip2 was co‐transfected with pRL‐TK into VSMCs treated with Ang II. pRL‐TK, which encodes Renilla luciferase, was used as an endogenous control. After 24 h of transfection, firefly and Renilla luciferase activities were detected via the Dual‐Luciferase Reporter Assay System (Yeasen, Shanghai, China, 11405ES60). Nrip2 promoter activity was calculated as the ratio of firefly luciferase activity to Renilla luciferase activity.

### Measurement of Mitochondrial Respiratory

Mitochondrial respiratory in VSMC was analysed using a mitochondrial function detection system (Orobors, Oxygraph‐2k, Austria). Briefly, VSMCs were cultured in a medium supplemented with 100 nmol L^−1^ Ang II and treated with or without Nrip2 plasmids for 24 h. Untreated cells (Con) served as the non‐Ang II control for baseline OCR comparison. The OCR was monitored in real time and normalized to total cellular protein content (measured via BCA assay). Values are expressed as pmol O_2_/min/mg protein, with sequential treatments with oligomycin (ATP synthase inhibitor), FCCP (mitochondrial uncoupler), and rotenone/antimycin A (respiration inhibitor) to evaluate OCR from proton leak, maximum respiration capacity, and nonmitochondrial respiration, respectively. OCR was measured multiple times at 10 intervals at each stage, and average values were determined.

### Transcriptomics Analysis

We prepared 5 × 10^5^ cells cultured in an Ang II‐treated medium, with or without Nrip2 plasmids. This was followed by the extraction of RNA and preparation of samples for mRNA isolation, mRNA fragmentation, cDNA synthesis, end repair, and adaptor ligation. The PCR system and program were configured to amplify the product. Single‐stranded PCR products were produced via denaturation. The reaction system and program for circularization were subsequently configured. Single‐stranded cyclized products were produced, while uncyclized linear DNA molecules were digested. Single‐stranded circular DNA molecules were replicated via rolling cycle amplification, and a DNA nanoball (DNB) containing multiple copies of DNA was generated. Sufficient‐quality DNBs were then loaded into patterned nanoarrays using a high‐intensity DNA nanochip technique and sequenced through combinatorial probe‐anchor synthesis.

### Statistical Analysis

Continuous data are expressed as mean ± standard error of the mean (SEM). the distribution of continuous variables was rigorously evaluated prior to selecting appropriate statistical tests. Normality was formally assessed using the Shapiro‐Wilk test for sample size less than 50 and the Kolmogorov‐Smirnov test for sample size more than 50. Only variables satisfying the assumptions of normality (*p* >0.05) were analyzed using parametric tests (Student's t‐test for equal variances or unequal variance t‐test for unequal variances, as confirmed by Levene's test). Variables violating normality assumptions were subjected to non‐parametric alternatives (Mann‐Whitney U test). When comparing three or more experimental conditions, one‐way or two‐way analysis of variance (ANOVA) was employed as appropriate, followed by post‐hoc tests with correction for multiple comparisons. Survival analysis was performed using the Kaplan‐Meier method, with the log‐rank test, and plotted with 95% confidence intervals. All statistical analyses were performed using SPSS version 25.0 (IBM Corp.) and GraphPad Prism version 8.0 (GraphPad Software). A two‐sided *p*‐value < 0.05 was considered statistically significant throughout the study.

## Conflict of Interest

The authors declare no conflict of interest.

## Supporting information



Supporting Information

## Data Availability

The prospective population data underlying this article were accessed from the UK Biobank (https://www.ukbiobank.ac.uk, application number:84352). The derived data generated in this research will be shared on reasonable request to the corresponding author.
