# Supplementary material for: Leptin Aggravates Thoracic Aortic Dissection Through Impairment of Energy Metabolism in Nrip2+ Smooth Muscle Cells
Source: Adv Sci (Weinh). 2025 Jul 16;12(38):e02027. doi: 10.1002/advs.202502027 (PMC12520536; doi:10.1002/advs.202502027)
Supplement: Supplementary file 1 — Supporting Information [file ADVS-12-e02027-s001.pdf]

ADVANCED SCIENCE
Open Access

Supporting Information

for *Adv. Sci.*, DOI 10.1002/advs.202502027

Leptin Aggravates Thoracic Aortic Dissection Through Impairment of Energy Metabolism in Nrip2^{+} Smooth Muscle Cells

Ling Chen, Yujie Zhu, Xi Yang, JiangBin Wu, Keyuan Chen, Wuqing Huang, Lu Fang, Qi Zhang, Jie Chen, Jianing Gao, Huanhuan Cao, Meifang Wu, Zhihuang Qiu, Yuling Zhang, Yue Shen, Qiuyu Huang, Zhiyong Lin, Lemin Zheng and Liangwan Chen**

SUPPLEMENTARY MATERIAL

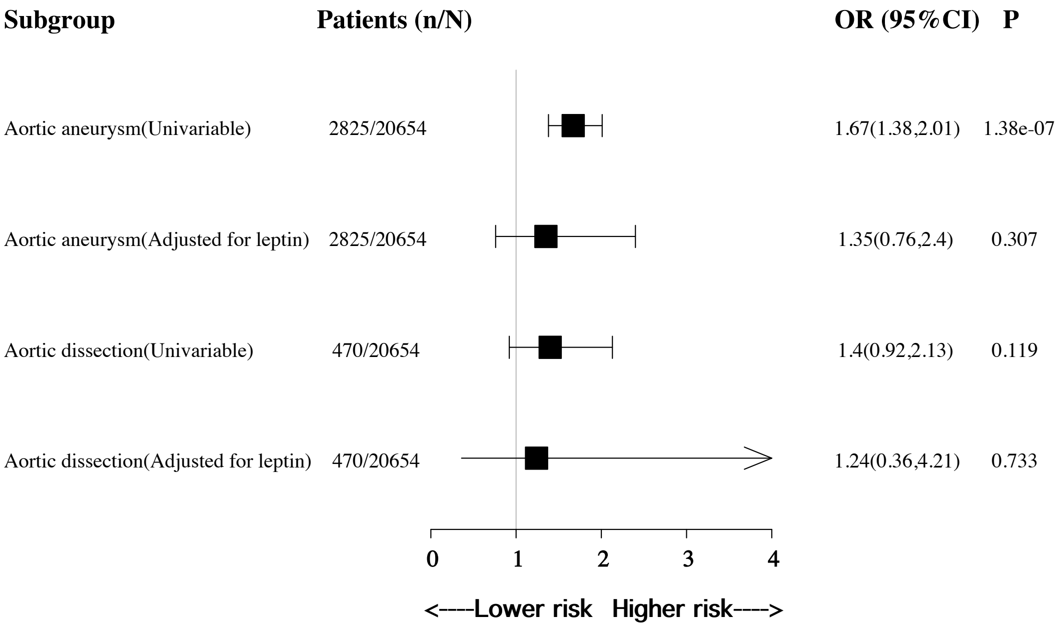

Supplementary Figure 1. Mendelian-randomization (MR) analysis reinforced leptin's tight association with BMI, revealing a robust link between BMI and aortic aneurysm (AA) risk (p=1.38E-07). Nevertheless, this correlation was mitigated (p=0.307) upon considering leptin's impact.

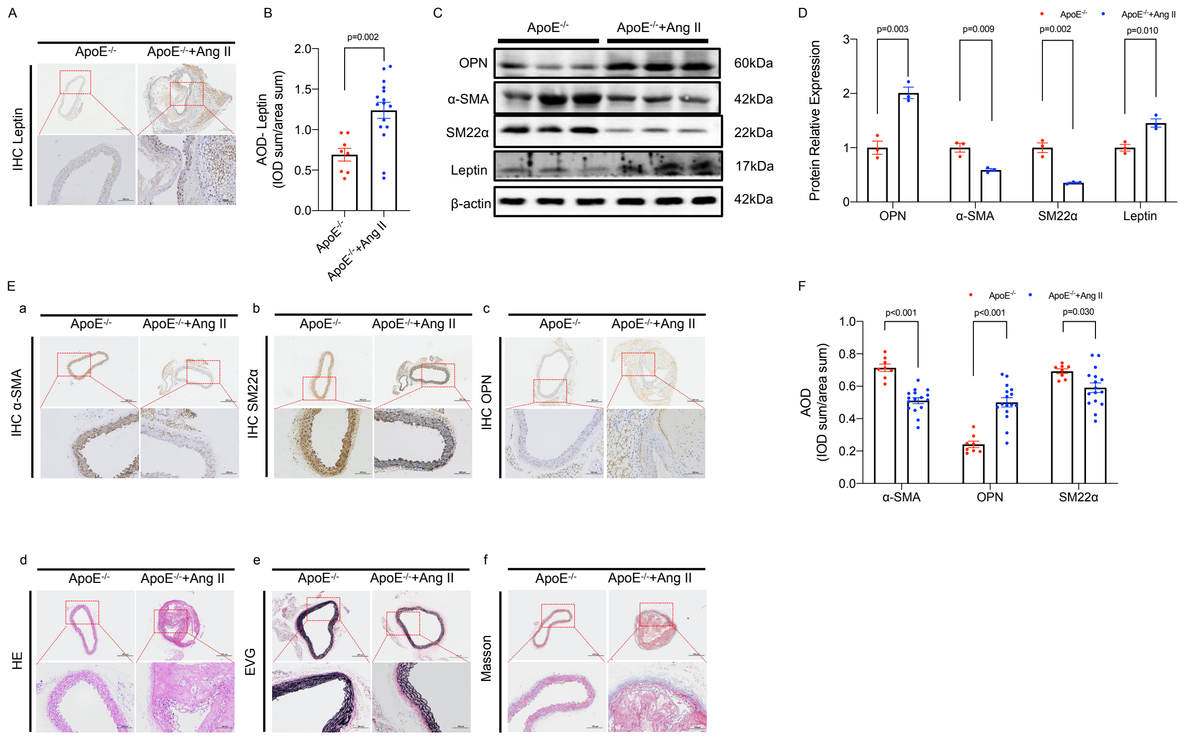

Supplementary Figure 2. Leptin exacerbates aortic pathology in an Ang II-induced ApoE$^{-/-}$ mouse model.

(A) Immunohistochemical detection of leptin in aortic sections. Scale bars: 300 µm (overview), 100 µm (high magnification). (B) Quantification of leptin immunohistochemical staining intensity (AOD: average optical density; IOD sum/area sum; n=8 ApoE$^{-/-}$, n=16 ApoE$^{-/-}$+Ang II). (C) Representative western blots of OPN (60 kDa), α-SMA (42 kDa), SM22α (22 kDa), leptin (17 kDa), and β-actin (42 kDa; loading control) in aortic tissues. (D) Densitometric analysis of protein expression normalized to β-actin (n=6/group). (E) Representative images of immunohistochemical staining for α-SMA (a), SM22α (b), and OPN (c), alongside histopathological evaluation using hematoxylin and eosin (HE; d), Elastic Van Gieson (EVG; e), and Masson's trichrome (f). Scale bars: 300 µm (overview), 100 µm (high magnification). (F) Quantification of α-SMA, SM22α, and OPN staining intensity

(AOD; n=8 $ApoE^{-/-}$, n=16 $ApoE^{-/-}$+Ang II). Data represent means ± SEM. Statistical significance was determined by Student's t-test (Figs B) or multiple t-tests (Figs D, F).

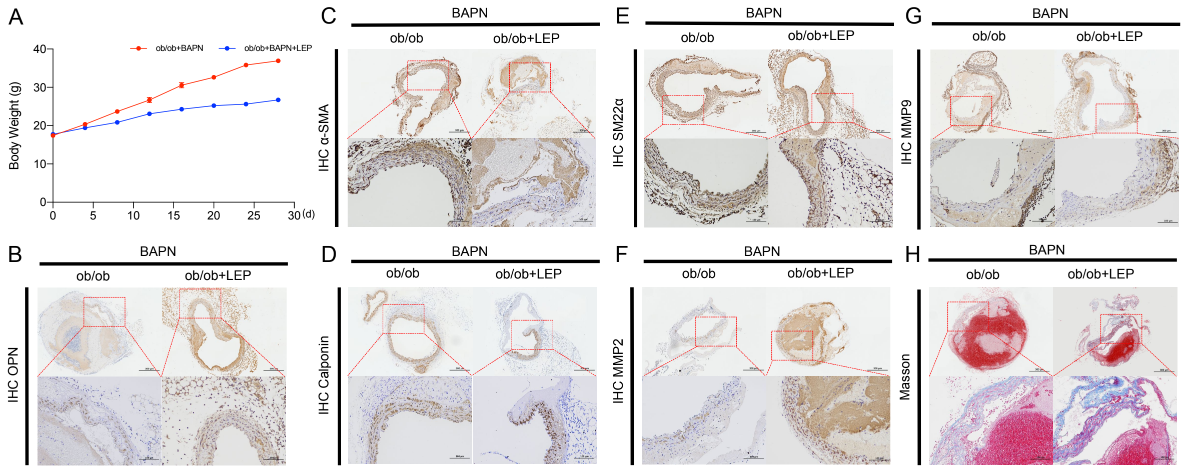

Supplementary Figure 3. Effects of leptin supplementation on body weight and vascular smooth muscle cell phenotype.

A) Body weight trajectories in ob/ob mice receiving BAPN alone (ob/ob+BAPN) versus those co-treated with leptin (ob/ob+BAPN+LEP) (n=16 per group). B-H) Macroscopic images of mouse aorta sections stained with immunohistochemical staining[antibodies against OPN (B),α-SMA (C), Calponin (D), SM22α (E), MMP2 (F), and MMP9 (G)] and Masson(H)(n=16; Scale bars: 300 μm (overview), 100 μm (insets); n=16 per group.

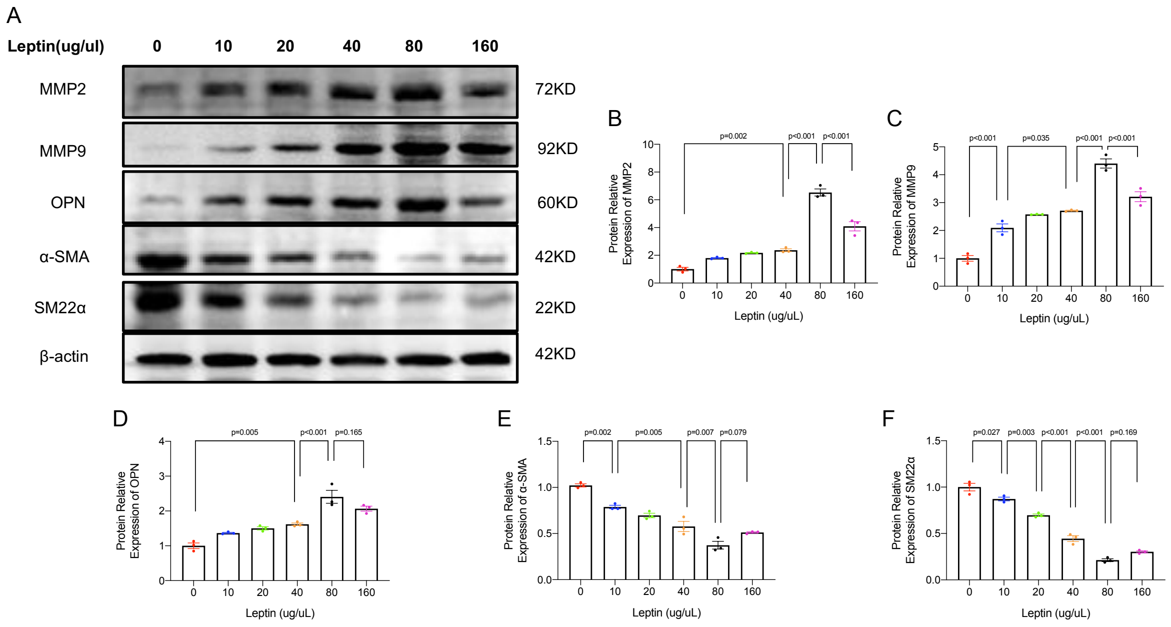

Supplementary Figure 4. Expression of MMP2/9, OPN, α-SMA, and SM22α in SMCs treated with different concentrations of leptin *in vitro* (n=3). Data are presented as means ± SEM. Comparisons across leptin concentrations were analyzed by one-way ANOVA followed by appropriate post hoc correction for multiple comparisons (Figs B–F).

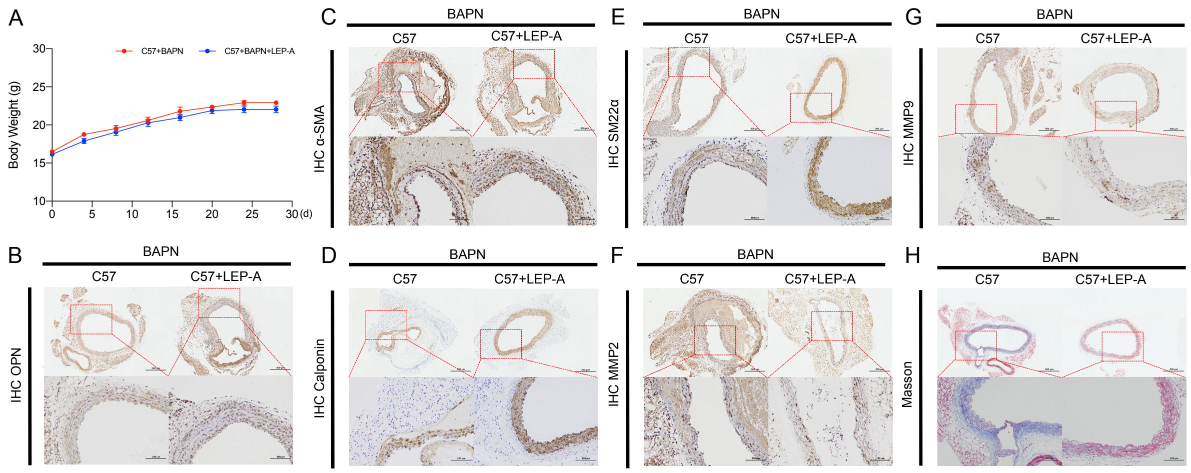

Supplementary Figure 5. Leptin antagonist (R128Q/LEP-A) attenuates vascular smooth muscle cell phenotypic transitions .

(A) Body weight trajectories in BAPN-induced TAD mice treated with saline (C57+BAPN) versus leptin antagonist (C57+BAPN+LEP-A) (n=16 per group). (B-H) Representative aortic sections from BAPN-treated mice: (B-G) Immunostained for phenotypic markers [Osteopontin (OPN; B), α-smooth muscle actin (α-SMA; C), Calponin (D), SM22α (E)] and extracellular matrix remodeling enzymes [matrix metalloproteinase-2 (MMP2; F), MMP9 (G)]; (H) Masson’s trichrome staining depicting connective tissue architecture. Scale bars: 300 μm (overview), 100 μm (insets); n=16 per group.

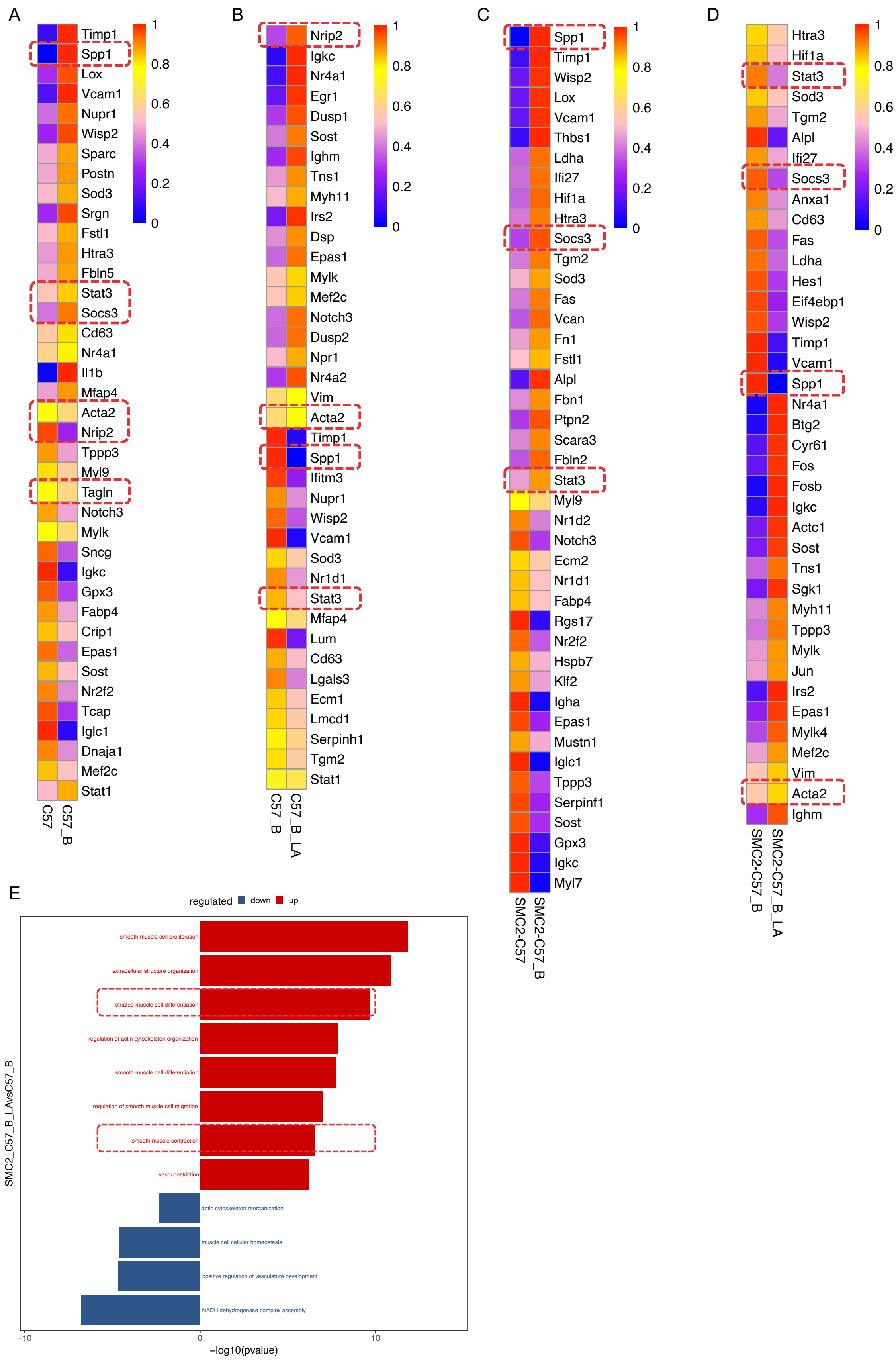

Supplementary Figure 6. Screen of differentially expressed genes (DEGs).

(A) The top DEGs between C57 and C57+BAPN group. (B) The top DEGs between C57+BAPN and C57+BAPN+LEP-A group. (C) The top DEGs of SMC cluster 2 between C57 and C57+BAPN group. (D) The top DEGs of SMC cluster 2 between C57+BAPN and C57+BAPN+LEP-A group. (E) Gene Ontology enrichment analysis of SMC cluster 2 between C57+BAPN and C57+BAPN+LEP-A group.

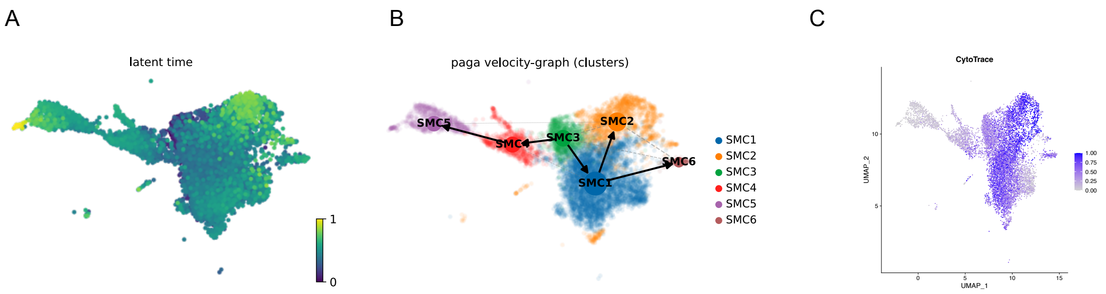

Supplementary Figure 7. Single-cell trajectory analysis of vascular smooth muscle cell differentiation dynamics.

(A) Pseudotime mapping illustrating developmental progression across VSMC clusters, where values (0–1) represent early to late differentiation stages. (B) Partition-based graph abstraction (PAGA) network overlaid on UMAP coordinates, with solid and dashed edges denoting strong and weak differentiation connections, respectively. (C) Cellular entropy/CytoTrace scores projected onto clustered UMAP embeddings, quantifying differentiation potential across subpopulations. The higher the entropy value /CytoTrace score, the higher the cell stemness and the greater the differentiation potential.

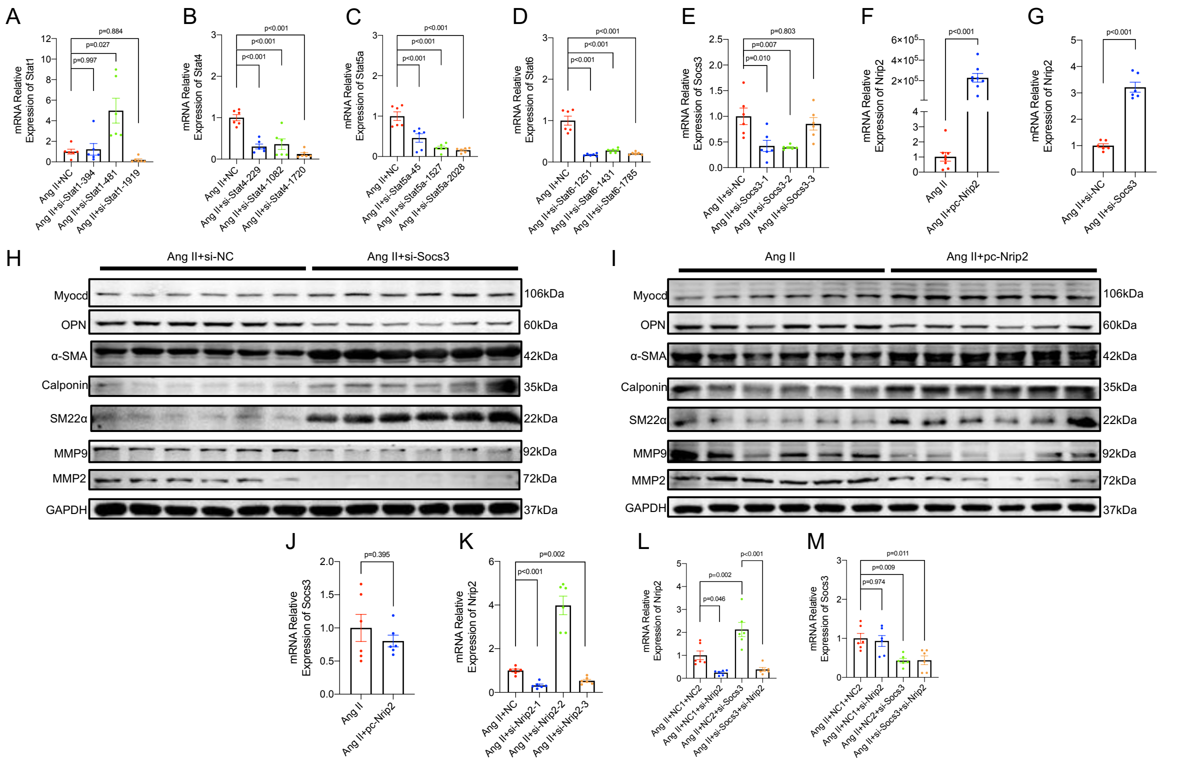

Supplementary Figure 8. Validation of siRNA efficiency and transcriptional regulation in vascular smooth muscle cells.

(A–D) Stat1 (A), Stat4 (B), Stat5a (C), and Stat6 (D) mRNA expression following transfection with gene-specific siRNAs. (E) Socs3 mRNA levels after Socs3 siRNA treatment. (F) Nrip2 expression in VSMCs transfected with Nrip2 plasmids. (G) Nrip2 mRNA levels following Socs3 siRNA transfection. (H) Representative immunoblots of phenotypic markers and MMP2/9 in Socs3 siRNA-treated VSMCs. (I) Western blot analysis of phenotypic markers and MMP2/9 after Nrip2 plasmid transfection. (J) Socs3 mRNA expression post-Nrip2 plasmid transfection. (K) Nrip2 mRNA levels in VSMCs treated with Nrip2 siRNA versus negative control (NC). (L) Nrip2 and (M) Socs3 mRNA expression in VSMCs co-transfected with Nrip2 and Socs3 siRNAs. Data represent mean ± SEM (n=6). Statistical analysis: One-way ANOVA with post-hoc correction (Figs A–E, K–M); Student's t-test (Figs G, J); Welch's t-test (Fig F). NC, negative control.

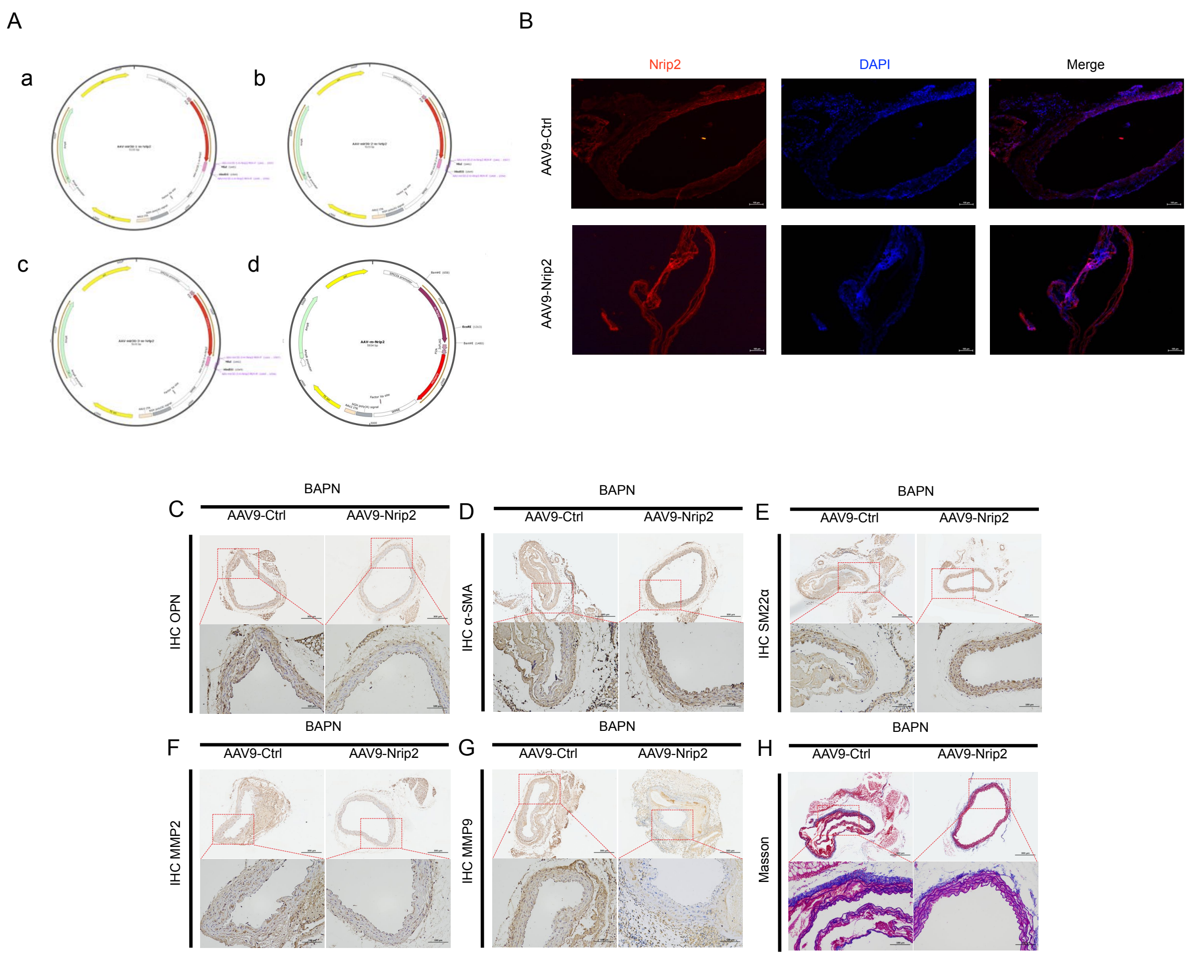

Supplementary Figure 9. AAV-mediated Nrip2 overexpression suppresses vascular smooth muscle cell phenotypic transition in mouse aortas.

(A) Schematic representation of AAV9-shNrip2 (a-c) and AAV9-Nrip2 (d) constructs. (B) Immunofluorescence detection of Nrip2 (red) with nuclear counterstaining (DAPI, blue) in aortic sections following AAV9-Ctrl or AAV9-Nrip2 treatment. Scale bar: 100 μm. (C-H) Representative immunohistochemical staining for osteopontin (OPN; C), α-smooth muscle actin (α-SMA; D), SM22α (E), matrix metalloproteinase-2 (MMP2; F), MMP9 (G), and Masson's trichrome staining for collagen deposition (H) in aortic tissues (n=10). Scale bars: 300 μm (overview), 100 μm (inset).

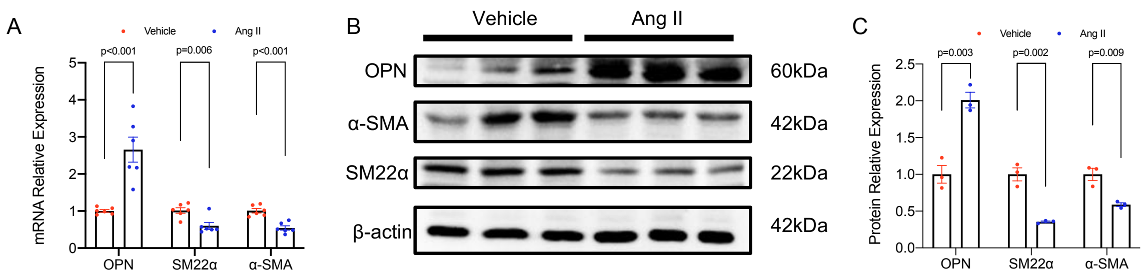

Supplementary Figure 10. Angiotensin II modulates phenotypic marker expression in vascular smooth muscle cells *in vitro*.

(A) mRNA expression levels of OPN, α-SMA, and SM22α in SMCs treated with Ang II. (B) Representative immunoblots and (C) quantitative analysis of OPN, α-SMA, and SM22α protein expression. Data represent mean ± SEM (n=3). Statistical analysis: Multiple t-tests (Fig A); Mann-Whitney U test (Figs B, C).

Supplementary Table 1. Baseline information of the study population, stratified by body mass index.

Characteristics	Underweight (n=2612)	Normal weight (n=162115)	Overweight (n=211643)	Obesity (n=121897)	P value*
**Age**	55.94±8.17	56.18±8.23	57.51±8.06	57.36±7.89	<0.001
**Sex**					<0.001
Male	537(20.56)	56559(34.89)	111879(52.86)	57665(47.31)	
Female	2075(79.44)	105556(65.11)	99764(47.14)	64232(52.69)	
**Ethnic background**					<0.001
Bristish	2254(86.29)	143034(88.23)	187428(88.56)	106909(87.70)	
Others	358(13.71)	19081(11.77)	24215(11.44)	14988(12.30)	
**Townsend deprivation index**	1.64±0.49	1.74±0.44	1.73±0.45	1.65±0.48	<0.001
**Physical activity (summed MET-min/week)**	2797±2765	2839±2726	2695±2737	2316±2620	<0.001
**Smoking status**					<0.001
Never	1473(56.39)	95492(58.90)	112901(53.34)	61886(50.77)	
Previous	539(20.64)	47696(29.42)	76061(35.94)	47409(38.89)	
Current	586(22.43)	18300(11.29)	21603(10.21)	11805(9.68)	
Unknown	14(0.54)	627(0.39)	1078(0.51)	797(0.66)	
**Alcohol intake frequency**					<0.001
Daily	573(21.94)	36596(22.57)	45254(21.38)	18631(15.28)	
Often	968(37.06)	80556(49.69)	106793(50.46)	54944(45.07)	

Seldom	653(24.31)	32885(20.29)	43890(20.74)	35496(29.12)	
Never	403(15.42)	11770(7.26)	15282(7.22)	12489(10.25)	
Unknown	15(0.57)	308(0.19)	424(0.20)	337(0.28)	
**Comorbidity**					
Hypertension	640(24.50)	54121(33.38)	106582(50.36)	80262(65.84)	<0.001
High cholesterol	358(13.71)	33110(20.42)	66486(31.41)	46996(38.55)	<0.001
Diabetes	40(1.53)	3393(2.09)	10223(4.83)	16112(13.22)	<0.001
Coronary heart diseases	71(2.71)	4520(2.79)	11491(5.43)	10510(8.62)	<0.001
**Outcome**					
Aortic aneurysm and dissection	19	891	1865	1246	
Follow up, person-years	2098418.663	32645.51781	2727681.584	1555645.737	
Incidence rate, per 1000 person-years	0.42	0.58	0.68	0.8	<0.001

Data is shown as [n (%) or mean±σ]; * Chi-square test or Fisher's exact test.

Supplementary Table 2. Follow-up data of TAD patients related to Figure 1A.

**Characteristics**	**Underweight (n=48)**	**Normal weight (n=268)**	**Overweight (n=517)**	**Obesity (n=184)**	**P value***
**Age**	50.73±14.28	54.06±12.83	54.09±11.17	47.21±11.37	0.017
**Sex**					0.001
Male	31(64.58)	194(72.39)	422(76.45)	131(87.92)	
Female	17(35.42)	72(26.87)	130(23.55)	18(12.08)	
**Comorbidity**					
Hypertension	20(41.67)	160(59.70)	440(78.72)	132(88.59)	<0.001
Diabetes	3(6.25)	4(1.49)	12(2.13)	4(2.68)	0.23

Data are shown as [n (%) or mean±σ]; * p values were calculated by ANOVA, Chi-square test or Fisher's exact test.

Supplementary Table 3. Hazard ratios of body mass index with incidence of aortic aneurysm and dissection related to Figure 1C.

**Characteristics**	**No. of individuals**	**No. of outcomes**	**Patients(n/N)**	**Crude**			**Adjusted**		
				**HR**	**95%CI**	**P-value**	**HR**	**95%CI**	**P-value**
Normal weight	162115	891	891/162115		Ref			Ref	
Underweight	2612	19	19/2612	1.37	0.88–2.17	0.165	1.7	1.00–2.89	0.050
Overweight	211643	1865	1865/211643	1.61	1.49–1.75	<0.001	1.1	1.01–1.22	0.025
Obesity	121897	1246	1246/121897	1.89	1.73–2.06	<0.001	1.33	1.20–1.47	<0.001

Adjusted for age, sex, ethnic background, Townsend Deprivation Index, physical activity, smoking, alcohol intake, and comorbidities. CI, confidence interval; HR, hazard ratio

Supplementary Table 4. MR analysis between body mass index and aortic aneurysm or aortic dissection related to Supplementary Figure 1.

**Subgroup**	**No. of cases**	**No. of control**	**Patients(n/N)**	**OR**	**CI**	**P-value**
Aortic aneurysm (Univariable)	2825	20654	2825/20654	1.67	1.67(1.38,2.01)	1.38E-07
Aortic aneurysm (Adjusted for leptin)	2825	20654	2825/20654	1.35	1.35(0.76,2.4)	0.307
Aortic dissection (Univariable)	470	20654	470/20654	1.4	1.4(0.92,2.13)	0.119
Aortic dissection (Adjusted for leptin)	470	20654	470/20654	1.24	1.24(0.36,4.21)	0.733

CI, confidence interval; MR, Mendelian randomization; OR, odds ratio.

Supplementary Table 5. Sequence of siRNAs.

	**Sense**	**Anti-Sense**
si-Socs3-1	GUAUGAUGCUCCACUUUAAUU	UUAAAGUGGAGCAUCAUACUG
si-Socs3-2	CAGCAUCUUUGUCGGAAGAUU	UCUUCCGACAAAGAUGCUGUU
si-Socs3-3	GCUUCGACUGUGUACUCAAUU	UUGAGUACACAGUCGAAGCGG
si-Nrip2-1	GCUGUAGAGGUCAAAGUUACC	UAACUUUGACCUCUACAGCCA
si-Nrip2-2	GGUAGAACAGCUGGAGUUAGA	UAACUCCAGCUGUUCUACCUG
si-Nrip2-3	GCUCUGCUAGUCAACUGCAAG	UGCAGUUGACUAGCAGAGCUG
si-Stat1-1	CACUGUGAUGUUAGAUAAAUU	UUUAUCUAACAUCACAGUGUU
si-Stat1-2	GCUGUUACUUUCCCAGAUAUU	UAUCUGGGAAAGUAACAGCUG
si-Stat1-3	GACCCUAGAAGAAUUACAAGA	UUGUAAUUCUUCUAGGGUCUU
si-Stat4-1	CGUCCAUUGACAAGAAUGUUU	ACAUUCUUGUCAAUGGACGUU
si-Stat4-2	CAUGGGAUUUGUUAGUAAAUU	UUUACUAACAAAUCCCAUGAU
si-Stat4-3	GCUAUUGAUUCACAAUCUAAA	UAGAUUGUGAAUCAAUAGCAG
si-Stat5a-1	AGGUCUUUGCCAAGUAUUAUU	UAAUACUUGGCAAAGACCUCG
si-Stat5a-2	GCCAGAUGCAAGUGUUGUAUU	UACAACACUUGCAUCUGGCGC
si-Stat5a-3	CGCUCAACAUGAAAUUCAAGG	UUGAAUUUCAUGUUGAGCGCU
si-Stat6-1	CCAAGACAACAACGCCAAAUU	UUUGGCGUUGUUGUCUUGGUU

si-Stat6-2	AGACCUGUCCAUUCGCUCAU	UGAGCGAAUGGACAGGUCUUU
si-Stat6-3	AGAAGAUCUUCAACGACAACA	UUGUCGUUGAAGAUCUUCUGG

Supplementary Table 6. Primers of target genes.

Primers	Forward 5'-3'
MMP2-F(homo)	CCACAGCCAACTACGATGATG
MMP2-R(homo)	CTCCTGAATGCCCTTGATGTC
MMP9-F(homo)	TTGACAGCGACAAGAAGTGG
MMP9-R(homo)	CCTCAGTGAAGCGGTACATAG
SM22α-F(homo)	AGAATGATGGGCACTACCGTG
SM22α-R(homo)	CTGTTGCTGCCCATCTGAAG
α-SMA-F(homo)	CCTGACTGAGCGTGGCTATT
α-SMA-R(homo)	GCCCATCAGGCAACTCGTAA
OPN-F(homo)	GAAGTTTCGCAGACCTGACAT
OPN-R(homo)	GTATGCACCATTCAACTCCTCG
MYOCD-F(homo)	ATCAATGAACTCACCTGGAAACTC
MYOCD-R(homo)	GCGGCTTCTTCTCTGAACAG
Calponin-F(homo)	GCTGGAGAACATCGGCAACT
Calponin-R(homo)	GGACTGCACCTGTGTATGGT
Leptin-F(homo)	TCCTCACCAGTATGCCTTCC
Leptin-R(homo)	TCTGTGGAGTAGCCTGAAGC
β-ACTIN-F(homo)	TGACGTGGACATCCGCAAAG
β-ACTIN-R(homo)	CTGGAAGGTGGACAGCGAGG
MMP2-F(mus)	AGATTGACGCTGTGTATGAGG

MMP2-R(mus)	TGTCTTCTTGTTCTTACTCCAGTT
MMP9-F(mus)	ATGTCACTTTCCCTTCACCTTC
MMP9-R(mus)	TGCCGTCCTTATCGTAGTCA
SM22α-F(mus)	GAAGGTGCCTGAGAACCCAC
SM22α-R(mus)	TGCTGCCATATCCTTACCTTCA
a-SMA-F(mus)	TCCCTGGAGAAGAGCTACGAAC
a-SMA-R(mus)	GACAGGACGTTGTTAGCATAGAG
OPN-F(mus)	AGCAAGAAACTCTTCCAAGCAA
OPN-R(mus)	GTGAGATTCGTCAGATTCATCCG
MYOCD-F(mus)	TTCAACACCACTGAGCAATACC
MYOCD-R(mus)	TCTGACACCTTGAGATCATCCA
Calponin-F(mus)	CCTACGGCTTGTCTGCTGAA
Calponin-R(mus)	CCGTCTTTGAGGCCATCCAT
Leptin-F(mus)	TCACACACGCAGTCGGTATC
Leptin-R(mus)	ACATTTTGGGAAGGCAGGCT
Nrip2-F(mus)	CAGCAGCGCCAACTCAAAC
Nrip2-R(mus)	GGCGTCTTTGGATCACACTGT
stat1-F(mus)	TGACGACCCTAAGCGAACTG
stat1-R(mus)	AGACATGGGAAGCAGGTTGT
stat4-F(mus)	TACCTTCTGGACTTGGCTTGA
stat4-R(mus)	GATGGCTCTCACTGAATCTTAACA

stat5a-F(mus)	CTGGATGAGAGCATGGATGTTG
stat5a-R(mus)	TTCTAGCGGAGGTGAAGAGAC
stat6-F(mus)	GGACTGCTTCCAGAGCACTT
stat6-R(mus)	AGACAGCGTTTGGTGAGGTC
Ndufb8-F(mus)	GCGAGTGGAAGACTACGAGC
Ndufb8-R(mus)	TCGGTTCACCCCAGTTCATC
Sdhb-F(mus)	AGTGCGGACCTATGGTGTTG
Sdhb-R(mus）	AGACTTTGCTGAGGTCCGTG
Mtco1-F(mus)	GTTCTATCAATGGGAGCAGTGTT
Mtco1-R(mus）	TCTGAGTAGCGTCGTGGTATT
Uqcrc2-F(mus)	ATGCCACCTTCTACCGTCCT
Uqcrc2-R(mus）	CACTCGCTGCCATTGACTTC
atp5a1-F(mus)	GACGCCCTCGGTAATGCTAT
atp5a1-R(mus）	TATCCACAGCCTTGATGCCG

Supplementary Table 7. The ELISA kits and antibodies

Reagent Name	Brand	Catalog Number
Leptin (ELISA)	Cloud-Clone Corp	SEA084Hu
Leptin (ELISA)	Cloud-Clone Corp	MEA084Mu
Leptin(Antibody)	abcam	ab16227
SM22α/ TAGLN2(Antibody)	abcam	ab121146
SM22α/ TAGLN3(Antibody)	abcam	ab14106
α-SMA(Antibody)	abcam	ab5694
Osteopontin/OPN(Antibody)	abcam	ab214050
Calponin(Antibody)	abcam	ab46794
MYOCD(Antibody)	affinity	AF0023
MYOCD(Antibody)	Thermo Fisher Scientific	PA5-100775
MMP9(Antibody)	abcam	ab76003
MMP2(Antibody)	abcam	ab92536
MMP2(Antibody)	abcam	ab97779
Nrip2(Antibody)	proteintech	17704-1-AP
Socs3(Antibody)	proteintech	14025-1-AP
GAPDH(Antibody)	abcam	ab181602
Tubulin(Antibody)	Beyotime	AF1216

β-actin(Antibody)	abcam	ab115777

Supplementary Methods

ELISA

Serum biomarker levels were determined using a double-antibody sandwich ELISA. Briefly, 25 µL of the test samples was collected and the test was performed according to the instructions of leptin ELISA kits (Cloud-Clone CORP, Wuhan, China, MEA084 and SEA084Hu).

Histology and immunohistochemistry

Aortas were fixed in 4% paraformaldehyde overnight, dehydrated with 20% sucrose embedded in wax, and sliced into 4-µm tissue sections. Sections were processed for elastic van Gieson (EVG), Masson trichrome, and haematoxylin and eosin (HE) staining. Sections were also immunostained using antibodies against α-SMA, OPN, SM22α, Calponin, MMP2, MMP9, and leptin, all at a 1:200 dilution. Positive immunoreactivity was visualized using DAB.

Protein extraction and western blotting

Proteins were extracted from thoracic aortas and MASMCs, separated using 4–12% SDS-PAGE, and transferred to a PVDF membrane. The membrane was blocked in 5% non-fat milk and incubated overnight at 4°C with primary antibodies. Protein bands were visualized via enhanced chemiluminescence using a chemiluminescence gel imaging system.

RNA interference

Assays with siRNAs, targeting mouse Socs3, Stat1, Stat4, Stat5a, and Stat6, were performed using Lipofectamine 3000 reagent. Primer sequence information is presented in Supplementary Table 5.

RNA isolation and real-time quantitative PCR (RT-qPCR)

Total RNA was isolated using TRIzol and then used for cDNA synthesis. mRNA expression was measured via RT-qPCR in a Real-time PCR System. β-actin was used as the housekeeping gene. Primer-related information is presented in Supplementary Table 6.

Animal models for AngⅡ induced TAD in ApoE$^{-/-}$ mice

Male ApoE$^{-/-}$ mice at 4 weeks of age were infused with angiotensin II (Ang II 1,000 ng/kg/min) using mini-osmotic pumps (Alzet Model 2004, DURECT Corporation) for 28 days to induce TAD.

Single-cell RNA sequencing tissue preparation and library construction

Fresh tissues were stored in tissue preservation solution on ice within postoperative 30 min. The specimens were washed with Hanks balanced salt solution three times, minced, and then digested using 3 mL tissue dissociation solution and Singleron PythoN® Tissue Dissociation System at 37°C for 15 min. The cell suspension was collected and filtered through a 40-μm sterile strainer. Subsequently, red blood cell lysis buffer (RCLB) was added, and the mixture (Cell: RCLB=1:2 [volume ratio]) was incubated at room temperature for 5–8 min. The mixture was then centrifuged at 300 *g* 4℃ for 5 min to remove the supernatant and resuspended with PBS. The sample was stained with trypan blue, and cell viability was evaluated microscopically.

Details of single-cell RNA-sequencing library preparation are previously described[1]. In brief, single-cell suspensions (2×105 cells/mL) with PBS (HyClone) were loaded onto a microwell chip using the Singleron Matrix® Single Cell Processing System. Barcoding beads were subsequently collected from the microwell chip, followed by reverse transcription of the mRNA captured by the barcoding beads to obtain cDNA, and PCR

amplification. The amplified cDNA was then fragmented and ligated with sequencing adapters. The scRNA-seq libraries were constructed according to the protocol of the GEXSCOPE® Single Cell RNA Library Kits (Singleron). Individual libraries were diluted to 4 nM, pooled, and sequenced on Illumina NovaSeq 6000 with 150-bp paired-end reads.

Primary analysis of raw read data (scRNA-seq)

Raw reads were processed to generate gene expression profiles using CeleScope v3.0.1 (Singleron Biotechnologies) with default parameters. Briefly, Barcodes and UMIs were extracted from R1 reads and corrected. Adapter sequences and poly-A tails were trimmed from R2 reads and the trimmed R2 reads were aligned against the GRCh38 (mm10) transcriptome using STAR(v2.6.1b). Uniquely mapped reads were then assigned to genes with FeatureCounts(v2.0.1). Successfully Assigned Reads with the same cell barcode, UMI, and gene were grouped together to generate the gene expression matrix for further analysis.

Quality control, dimension-reduction and clustering (Scanpy)

Scanpy v1.8.2 was used for quality control, dimensionality reduction and clustering under Python 3.7. For each sample dataset, we filtered expression matrix by the following criteria: 1) cells with a gene count less than 200 or with a top 2% gene count were excluded; 2) cells with a top 2% UMI count were excluded; 3) cells with mitochondrial content > 20% were excluded; 4) genes expressed in less than 5 cells were excluded. After filtering, 87169 cells were retained for the downstream analyses, with on average 1227 genes and 3074 UMIs per cell. The raw count matrix was normalized by total counts per cell and logarithmically transformed into normalized data matrix. Top 2000 variable genes were selected by setting flavor = ‘seurat’. Principle Component Analysis (PCA) was performed on the scaled variable gene matrix, and top 20 principle components

were used for clustering and dimensional reduction. Cells were separated into 40 clusters by using Louvain algorithm and setting resolution parameter at 1.2. Cell clusters were visualized by using Uniform Manifold Approximation and Projection (UMAP).

Cell-type recognition with Cell-ID

Cell-ID is multivariate approach that extracts gene signatures for each cell and perform cell identity recognition using hypergeometric tests (HGT)[2]. Dimensionality reduction was performed on normalized gene expression matrix through multiple correspondence analysis, where both cells and genes were projected in the same low dimensional space. Then a gene ranking was calculated for each cell to obtain most featured gene sets of that cell. HGT were performed on these gene sets against brain reference from SynEcoSys database, which contains all cell-type's featured genes. Identity of each cell was determined as the cell-type has the minimal HGT p value. For cluster annotation, Frequency of each cell type was calculated in each cluster, and cell type with highest frequency was chosen as cluster’s identity. The cell type identification of each cluster was determined according to the expression of canonical markers from the reference database SynEcoSys® (Singleron Biotechnology). SynEcoSys® contains collections of canonical cell type markers for single-cell seq data, from CellMakerDB, PanglaoDB and recently published literatures.

To obtain a high-resolution map of SMCs, cells from the specific cluster were extracted and reclustered for more detailed analysis following the same procedures described above and by setting the clustering resolution as 0.4.

References

1. Wu F, Fan J, He Y, Xiong A, Yu J, Li Y, et al. Single-cell profiling of tumor heterogeneity and the microenvironment in advanced non-small cell lung cancer. Nature communications. 2021;12:2540.

2. Cortal A, Martignetti L, Six E, Rausell A. Gene signature extraction and cell identity recognition at the single-cell level with cell-id. Nature Biotechnology. 2021;39:1095-1102.
